# Hydrological regime of a continental river system predicts bacterial macroecological patterns

**DOI:** 10.1093/ismejo/wrag013

**Published:** 2026-02-02

**Authors:** Katalin Demeter, Domenico Savio, Alexander K T Kirschner, Georg H Reischer, Stoimir Kolarevic, Juraj Parajka, Julia Derx, Stefan Jakwerth, Christian Wurzbacher, Alfred P Blaschke, Robert L Mach, Günter Blöschl, Andreas H Farnleitner, Alexander Eiler

**Affiliations:** Research Group Microbiology and Molecular Diagnostics E166-5-3, Institute for Chemical, Environmental and Bioscience Engineering and Research Centre Water & Health E057-08, TU Wien, Gumpendorferstraße 1a, 1060 Vienna, Austria; Interuniversity Cooperation Centre Water and Health, waterandhealth.at; Research Group Microbiology and Molecular Diagnostics E166-5-3, Institute for Chemical, Environmental and Bioscience Engineering and Research Centre Water & Health E057-08, TU Wien, Gumpendorferstraße 1a, 1060 Vienna, Austria; Interuniversity Cooperation Centre Water and Health, waterandhealth.at; Department of Water Quality and Health, Faculty of Health Sciences, Karl Landsteiner University, Dr.-Karl-Dorrek-Straße 30, 3500 Krems an der Donau, Austria; Interuniversity Cooperation Centre Water and Health, waterandhealth.at; Department of Water Quality and Health, Faculty of Health Sciences, Karl Landsteiner University, Dr.-Karl-Dorrek-Straße 30, 3500 Krems an der Donau, Austria; Centre for Pathophysiology, Infectiology and Immunology, Institute for Hygiene and Applied Immunology, Water Microbiology Group, Medical University Vienna, Kinderspitalgasse 15, 1090 Vienna, Austria; Research Group Microbiology and Molecular Diagnostics E166-5-3, Institute for Chemical, Environmental and Bioscience Engineering and Research Centre Water & Health E057-08, TU Wien, Gumpendorferstraße 1a, 1060 Vienna, Austria; Interuniversity Cooperation Centre Water and Health, waterandhealth.at; Institute for Biological Research “Siniša Stanković” – National Institute of Republic of Serbia, University of Belgrade, Bulevar despota Stefana 142, 11060 Belgrade, Serbia; Institute of Hydraulic Engineering and Water Resource Management, Research Area Hydraulic Engineering and Water Quantity Management, TU Wien, Karlsplatz 13/222, 1040 Vienna, Austria; Interuniversity Cooperation Centre Water and Health, waterandhealth.at; Institute of Hydraulic Engineering and Water Resource Management, Research Area Hydraulic Engineering and Water Quantity Management, TU Wien, Karlsplatz 13/222, 1040Vienna, Austria; Interuniversity Cooperation Centre Water and Health, waterandhealth.at; Centre for Pathophysiology, Infectiology and Immunology, Institute for Hygiene and Applied Immunology, Water Microbiology Group, Medical University Vienna, Kinderspitalgasse 15, 1090 Vienna, Austria; Chair of Urban Water Systems Engineering, Technical University of Munich, Am Coulombwall 3, 85748 Garching, Germany; Interuniversity Cooperation Centre Water and Health, waterandhealth.at; Institute of Hydraulic Engineering and Water Resource Management, Research Area Hydraulic Engineering and Water Quantity Management, TU Wien, Karlsplatz 13/222, 1040 Vienna, Austria; Institute of Chemical, Environmental and Bioscience Engineering, TU Wien, Gumpendorferstraße 1a, 1060 Vienna, Austria; Institute of Hydraulic Engineering and Water Resource Management, Research Area Hydraulic Engineering and Water Quantity Management, TU Wien, Karlsplatz 13/222, 1040 Vienna, Austria; Research Group Microbiology and Molecular Diagnostics E166-5-3, Institute for Chemical, Environmental and Bioscience Engineering and Research Centre Water & Health E057-08, TU Wien, Gumpendorferstraße 1a, 1060 Vienna, Austria; Interuniversity Cooperation Centre Water and Health, waterandhealth.at; Department of Water Quality and Health, Faculty of Health Sciences, Karl Landsteiner University, Dr.-Karl-Dorrek-Straße 30, 3500 Krems an der Donau, Austria; Department of Biosciences, Centre of Biogeochemistry in the Anthropocene, Section for Aquatic Biology and Toxicology, University of Oslo, Blindernveien 31, 0371 Oslo, Norway; eDNA solutions AB, Sahlgrenska Science Park, Medicinaregatan 9E, 41390 Gothenburg, Sweden

**Keywords:** bacterial carbon assimilation, bacterial community composition, aquatic microbial ecology, metacommunity

## Abstract

Modeling bacterial dynamics in large river systems is crucial for predicting continental-scale ecosystem functioning under anthropogenic pressures. Although the River Continuum and Metacommunity concepts have provided theoretical frameworks, quantitative parameters necessary for microbial macroecological models remain scarce. Here, we present results from two whole-river surveys, conducted six years apart along 2600 km of the Danube River. Using bacterial secondary production, cell counts, and 16S ribosomal RNA (rRNA) gene amplicon sequencing, we quantified carbon, cell, phylotype, and diversity turnover along the river. Carbon incorporation per cell declined with water travel time by 6000–21 000 atoms per hour. Bacterial cells multiplied every eight days, resulting in four to six doublings during downstream transport. Growth responses at the level of individual phylotypes differed up to a hundredfold from these bulk community estimates. Bacterial diversity dynamics were dominated by phylotype turnover rather than phylotype loss. Turnover ranged from 0.92 to 0.96 along the river, indicating an almost complete replacement of phylotypes with 2%–11% of headwater-associated amplicon sequence variants (ASVs) persisting under base-flow conditions. Richness declined gradually downstream at a rate of ~0.13 ASVs per hour. Variations in bacterial secondary production, cell abundance, and observed ASVs were best explained by models combining hydrological and water quality parameters, whereas beta diversity followed a gradual development primarily structured by water travel time. Together, these results identify water travel time as the key integrative parameter governing microbial macroecological dynamics along large rivers, with environmental conditions fine-tuning local responses. These models can help predict changes in microbial diversity and functioning under anthropogenic alterations.

## Introduction

Microorganisms play an integral and often unique role in the functioning of ecosystems, thereby providing essential ecosystem services to human society. Gaining insight into the large-scale patterns of microorganisms—including their carbon, cell, species, and community turnover—is thus essential for predicting anthropogenic impacts on terrestrial and aquatic ecosystems. Macroecological studies on bacterial communities, including both experimental and large-scale surveys, have revealed that communities are assembled by ecological drift, dispersal-related processes, and local environmental conditions, in a process referred to as “species sorting” [1–4]. Even though these and other [5–8] studies have used theoretical frameworks (i.e. River Continuum and Metacommunity concepts) to make qualitative predictions on bacterial macroecological dynamics, the question of the relationship between bacterial diversity and function along continental drainage networks is still insufficiently addressed. Actual quantitative rates of diversity and functional changes due to anthropogenic impacts are largely missing.

In aquatic systems, water residence time (WRT) is considered to be a main parameter in determining diversity [7–9] and was shown to regulate functions such as carbon, cell, and biomass turnover [10–12]. Residence time refers to the average time water spends within a specific system (e.g. in a lake), whereas water travel time (WTT) or flow time refers to the duration water takes to travel a specific distance (e.g. along a river segment). Flow velocity is the speed at which water moves at a given point, and dendritic length describes the stream length in a branching river network. Furthermore, local environmental conditions are often considered paramount in shaping bacterial community composition [13–17] and microbial functioning, including bacterial secondary production (BSP) [10, 18], particularly in aquatic environments with long WRT (corresponding to long WTT and low flow velocity), such as lakes, soils, alluvial groundwater aquifers, and the open ocean.

With increasing river width and decreasing riparian and groundwater influence, species sorting is progressively considered to prevail over allochthonous inputs of bacterial cells due to dispersal, i.e. “mass effects,” in shaping the bacterioplankton diversity. For headwater streams, the generally high flow velocities and low WRT (short WTT) necessitate that the initial mass effects must exceed the rate of local extinction (export rate) to sustain cell densities as typically observed even in extremely pristine streams [6, 19]. Conversely, in large rivers where cell imports from tributaries are negligible, internal cell production must exceed the loss rates to explain the observed increasing cell densities [20–23]. Cell production is thereby directly related to BSP, the rate at which bacteria create new biomass by consuming organic matter. Hence, BSP is limited by autochthonous carbon fixation as well as allochthonous input of organic carbon sources and nutrients [10, 12]. The latter includes anthropogenic sources such as sewage, city runoff, or agricultural runoff. At a higher level, nutrient inputs, cell production, and losses—and therefore cell densities—are governed by the prevailing hydrological conditions, particularly WRT [10, 12, 18], which are highly modified by human activity. Mass effects from tributaries and diffuse sources can vary locally and over temporal scales [24], potentially disrupting gradual macroecological patterns.

Surface-runoff-induced riparian influence is mainly determined by precipitation events [25], whereas droughts represent conditions of minimum riparian influence. Consequently, hydrological and meteorological conditions in the catchment regulate both the internal production and the mass effects, thereby determining bacterial biomass as well as organic matter transformation and concentrations [26–29]. Although temporary fluctuations in the hydrological regime in response to meteorological events have been shown to affect the dynamics and observable distribution of bacterial taxa [30, 31], these observations have yet not been put into the context of macroecological dynamics. Quantitative models are needed to predict microbial dynamics, such as BSP, and cell, genotype, and community turnover, occurring in the hydrological path from the continents to the ocean in light of climate change and other anthropogenic pressures.

For modeling purposes, the effects of the hydrological regime (including WRT, WTT, dendritic distance, and flow velocities) on bacterial species and community turnover need to be understood in the context of bacterial production (i.e. cell division rates or doubling times) and the standing stock (i.e. cell abundances and biomass), and thus cell turnover rates. Typical bacterial concentrations in streams and rivers are reported to range from 10^5^–10^8^ cells per milliliter [20, 32] with daily cell division rates ranging from 0.1 to 2 [10, 21, 33, 34]. By calculating the doubling times (calculated as 1/division rate) under a given WTT or WRT, we can estimate the number of generations on which species sorting can act in a system [10]. Relating hydrological regimes with cell, species, and community turnover rates are essential for the modeling of temporal and spatial variations in microbiological water quality and nutrient budgets over continental scales. This is of particular importance, considering that rivers represent the major link from the continents to the ocean in the global water cycle and provide many ecosystem services such as drinking water by river bank filtration, sources for irrigation water, transport routes for shipping, and contaminant degradation.

**Figure 1:**
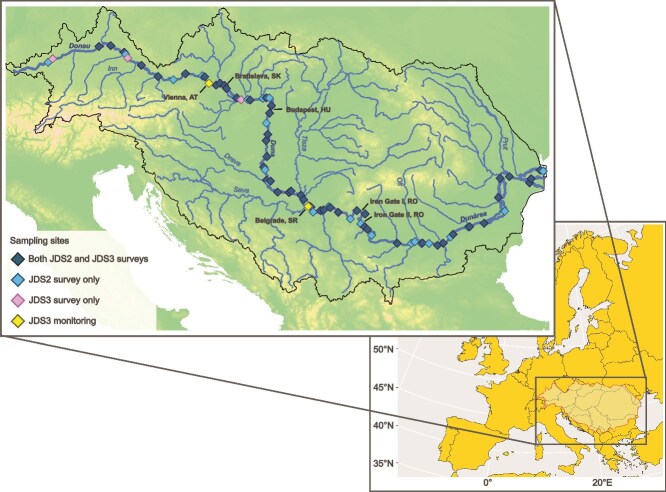
Map of the Danube River catchment illustrating the sampling sites during the Joint Danube Survey 2 (JDS2, taken from [7], sampling sites = 75), the Joint Danube Survey 3 (JDS3, sampling sites = 54) and the 1-year monthly monitoring campaign following JDS3 (*n* = 2). Country capitals along the Danube and the two largest dams along the river, namely, Iron Gate I and II, are depicted in black font. The map in the background shows the Danube River catchment within Europe.

Here, we hypothesize that changes in individual bacterial phylotypes as well as in diversity depend on the hydrological regime and bacterial generation times, which are, in turn, highly influenced by climate and anthropogenic modification to the continental drainage systems. As a study site, we selected a 2600 km–long stretch of the Danube River (Fig. 1), the second largest river in Europe by discharge and length, which drains a basin of ~801 000 km^2^ belonging to 19 countries [35]. We analyzed the datasets of two whole-river surveys conducted 6 years apart, supplemented by data from a monthly sampling campaign over 1 year at two sites. This unique setting allows us to study the upstream-to-downstream evolvement of bacterial diversity and functioning over a WTT of several weeks. We hypothesized that this long WTT, coupled with the warm water temperatures during the surveys, allows for sufficient bacterial cell divisions to observe species sorting. We estimated the number of bacterial cell divisions and the total cell production during the WTT based on measurements of bacterial secondary production and cell counts. Additionally, we analyzed bacterial diversity based on 16S rRNA gene sequences. Observations on the effect of discharge and anthropogenic factors, such as impoundments and wastewater treatment plants, complement this comprehensive study.

## Materials and methods

### Joint Danube Surveys: comprehensive ecological research campaigns

Within this study, we analyzed data from two large river surveys along the continental Danube River – namely, ‘Joint Danube Survey (JDS)’ 2 and 3 (Fig. S1) – conducted in the summers of 2007 and 2013. Using two surveys instead of one can improve the robustness and reliability of results, especially when dealing with complex phenomena. It can identify potential biases, inconsistencies, or limitations inherent in a single survey.

The overall purpose of the Joint Danube Surveys is to produce a comprehensive evaluation of the chemical and ecological status of the entire Danube River on the basis of the European Union Water Framework Directive (WFD), as well as the assessment of the microbiological status of the river [36]. Within the scope of JDS3 (2013), in total, >800 individual chemical, microbiological, ecotoxicological, radiological, and biological parameters were investigated [37–40]. During JDS2 (2007), over 280 individual parameters were determined [7, 20, 36]. JDS3 data on bacterial abundances, secondary production, and community dynamics are analyzed here for the first time, whereas JDS2 data were published previously [7] and are re-analyzed in this study. To complement our assessment of bacterial macroecological functioning, we have added a set of key environmental parameters to our analyses, including hydrological estimates, nutrient, and chlorophyll concentrations, from the vast JDS2 and 3 datasets.

### Experimental procedures during Joint Danube Survey 2

The study design and experimental procedures of Joint Danube Survey 2 for the results that are re-analyzed in the current study were published by Savio *et al*. [7]. In short, JDS2 was conducted between 15 August and 26 September 2007, with 75 sites sampled along the mainstream of the Danube River along its navigable way from river kilometer (rkm) 2600 near Ulm (Germany) to the river mouth at rkm 18 (Romania) (Fig. 1).

### Study sites during Joint Danube Survey 3

JDS3 was conducted between 13 August and 25 September 2013. The sampling design was built on insights gained from JDS1 and JDS2 [41], and its description goes beyond the scope of this manuscript [37]. For this study, we selected the water samples collected in the midstream at all 54 sampling stations along the longitudinal profile of the Danube River [37] (Fig. 1). This covers the shippable way from river kilometer (rkm) 2581 at Ulm/Böfinger Halde (Germany) to rkm 18 close to the river’s mouth at the Black Sea (Romania). Aside from the longitudinal snapshot study, two sites (downstream of the cities of Vienna and Belgrade, rkm 1919 and rkm 1159, respectively) were sampled monthly over ~1 year in 2014 (*n* = 27).

Geomorphological measures and the estimation of WTT are described in the Supplementary Information.

### Bacterial numbers, biometry, and bacterial secondary production

Total prokaryotic cell count (*TCC*) and mean cell volumes (m*V_c_*) were determined by fluorescence microscopy as described previously by Velimirov *et al*. [20], with the difference that for JDS3, cells were stained using Sybr Gold fluorescence dye (Invitrogen SYBR Gold, Thermo Fisher Scientific; MA, USA) and counted on a “Nikon Eclipse 80i” epifluorescence microscope, whereas for JDS2, Acridine orange (Merck, Darmstadt, Germany) staining dye and a “Leica Diaplan” epifluorescence microscope were used. Moreover, for JDS2, cells were categorized as coccus-, rod-, and vibrio-shaped cells for both the free-living (FL, size 0.2–3.0 μm) and particle-associated (PA, >3 μm) filter fractions [20], which were summed up (cell categories and filter fractions) to give estimates of the bulk samples for the current analysis. During JDS3, no separation of the two size fractions was made and cells were categorized as either “small” or “large cells” regarding counting and morphometry and summed up (cell categories) for data analysis.

Bacterial secondary production (*BSP*) was determined for the bulk microbial communities based on ^3^H-leucine incorporation as described previously [20]. For more details on calculations, see the “Formula collection” and Fig. S2 in the Supplementary Information.

### 16S rRNA gene amplicon sequencing and data analysis

Sample collection, DNA extraction, 16S rRNA gene amplicon library preparation, and paired-end sequencing are described in detail in the Supplementary Information. Community composition analysis was performed on fractioned samples for JDS2, FL and PA, whereas in JDS3, it was done on the bulk sample.

Previously published and deposited amplicon libraries for JDS2 (150 samples, 75 sites with two fractions each, no replicates) and JDS3 (675 samples covering the entire JDS3 sample set, including 66 technical and 329 biological replicate pairs) were downloaded from the National Center for Biotechnology Information (NCBI) Sequence Read Archive (accession number SRP045083 [7] and PRJNA835446 [42]). These two raw sequence datasets were processed separately. We removed adapter and primer sequences from the demultiplexed sequences using the CUTADAPT tool [43]. Sequences without matching primers were discarded. The R package dada2 ([44], version 1.8) was used for de-replication, denoising, and sequence pair concatenation. Taxonomy was assigned using the Bayesian classifier and the SILVA nonredundant database 138 [45, 46]. Next, the biological replicate pairs for the JDS3 dataset were merged and averaged to obtain the final amplicon sequence variant (ASV) table. Chloroplast, cyanobacterial, mitochondrial, eukaryotic, and archaeal ASVs were removed, resulting in 10 801 (JDS2) and 12 417 (JDS3) ASVs. Both the biological and the technical replicates showed high similarity [Fig. S1, e.g. median and range of the Bray–Curtis dissimilarity of the biological replicates in the sample set used for this study were 0.15 (0.10–0.59)]. Libraries with <3155 sequence reads were discarded, whereas the remaining libraries were randomly resampled with replacement in 50 consecutive repetitions using the “rrarefy”-function implemented in the R package “vegan.” After averaging the results from 50 repetitions and rounding, the mean number of sequence reads was 3152 reads per sample. The JDS3 sample set was then filtered for the sites of interest (in the longitudinal set: main stem only, midstream samples only, *n* = 54, in the temporal monitoring: Vienna and Belgrade, *n* = 27). All samples passing the above steps were used for JDS2 (FL *n* = 39, PA *n* = 47).

Alpha diversity was described by the count of observed ASVs. For the partitioning of beta diversity in regard to the respective turnover (i.e. species replacement between two sites) and nestedness (i.e. species loss between two sites) components, functions implemented in the R package “betapart” were used [47]. The Simpson dissimilarity is bounded between 0 and 1, where 0 means the two sites have the same composition (that is, they share all the species and the species have the same abundances), and 1 means the two sites do not share any species. As turnover was the main driver, we used Bray–Curtis dissimilarity index for modeling the variation in species composition among sites. This index was calculated using sequence read abundances that were first normalized to 1 for each sample using the “drarefy”-function implemented in the R-package “vegan” [48].

### Statistical analyses

Statistical analyses and plot generation were conducted in R, versions 3.6.2 to 4.5.1 [49]. Linear regression models were calculated using the “lm” function implemented in R, reporting an “adjusted R squared” as a measure for the “goodness of fit” and the standard error as a measure for variation. The covariation between environmental variables and the projections of bacterioplankton community samples in the Bray–Curtis-based nonmetric multidimensional scaling (nMDS) was calculated using the “envfit” function included in the R-package “vegan” [48]. Generalized additive models (gam, package “mgcv” [50]) with simple factor smoothers on travel time were fitted to a selection of ASVs, in terms of cell abundances, calculated from relative abundances and *TCC*. ASVs were selected based on a minimum ratio between maximum and minimum relative abundance of 100, a minimal maximum abundance of 1% in at least one dataset, and a minimal prevalence in at least 20 samples. The fitted curves were then differentiated, using the derivatives function implemented in the “gratia” package [51]‚ to estimate the net changes of each ASV. From the fitted derivative curves, we calculated the maximum absolute decrease or increase for each ASV.

### Data availability

All data of Joint Danube Surveys 1, 2, and 3 are publicly available via the official website of the International Commission for the Protection of the Danube River (ICPDR; http://www.icpdr.org/wq-db/), and the final scientific report in particular [37]. Selected data from JDS 1, 2, and 3 were published previously in several studies [36–38, 42, 52–55]. Raw sequence data are available on the NCBI Sequence Read Archive (accession number SRP045083 [7] for JDS2 and PRJNA835446 [42] for JDS3).

All data presented in the current study, such as rarefied ASV tables, corresponding taxonomy tables, derived alpha and beta diversity metrics, and metadata tables (hydrological, physicochemical, and other water quality parameters), are available on Zenodo (DOI: https://zenodo.org/records/18216076).

## Results and discussion

### Hydrological and water quality conditions during the Joint Danube Surveys 2 and 3

The Danube River surveys were conducted under contrasting hydrological regimes: mean flow conditions during the 2007 campaign (JDS2) and prevailing low flow conditions during the 2013 campaign (JDS3) (Fig. S3, [37]). The course of the Danube River is heavily modified by river regulation and damming predominantly in th eupper section (DE, AT), and with the two Iron Gate Reservoirs between the middle and lower section (RO, SRB, Fig. S1) being the most significant impoundments. This results in highly varying flow velocities along the hydrological path (ranging from 0.6 to 11.2 km h^−1^ during JDS2 and from 0.9 to 5.4 km h^−1^ during JDS3, Fig. S3).

Conducted during summer, water temperatures were on average 21.1°C during JDS2 and 20.9°C during JDS3 (Fig. S4). Nitrate concentrations showed an initially synchronous decrease (from 13.8 to 3.9 mg l^−1^ in JDS2 and from 12.0 to 3.9 mg l^−1^ in JDS3); a slight increase was observed after the Iron Gates for JDS2 (up to 7.3 mg l^−1^). Orthophosphate concentrations showed no clear longitudinal trends, though having lower concentrations during JDS2 (mean 0.11 mg l^−1^) than JDS3 (mean 0.15 mg l^−1^). Phytoplankton abundance, as indicated by chlorophyll-a measurements, showed stark regional differences, indicating significant point sources of nutrient input between the two major cities, Budapest and Belgrade (ranging from 0.7 to 30.6 μg l^−1^during JDS2 and from 0.3 to 16.0 μg l^−1^ during JDS3, Fig. S5, [37]). Further environmental variables (conductivity, pH, dissolved oxygen, total suspended solids, dissolved organic carbon, and the fecal indicator *Escherichia coli*) are shown in the Supplementary Materials (Figs S4 and S5).

### Trends in bacterial cell concentrations

**Figure 2:**
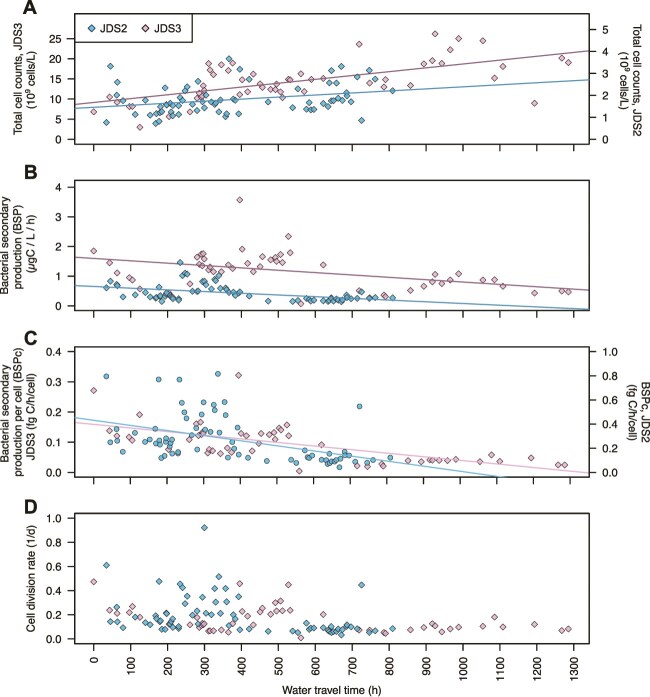
Trends in prokaryotic cell counts and metabolic parameters along the Danube River with increasing travel time (*ttcum*). (A) Prokaryotic cell concentration (total cell counts; *TCC*), (B) Bulk bacterial secondary production (BSP), (C) Bacterial secondary production per cell (*BSPc*) and (D) daily cell division rates (*CD_d_*). Descriptive statistics are shown in Table 1, whereas regression statistics and sample numbers are shown in Table 2. (data for JDS2 were taken from [20]). *n*(JDS2) = 71, *n*(JDS3) = 54.

Total bacterial cell concentrations—or “total cell counts” (*TCC*), enumerated using epifluorescence microscopy—showed a clear overall increasing trend along the river (Tables 1 and 2; Fig. 2A, Fig. S6). The ~10-fold difference in cell concentrations between the two surveys is likely attributable to different hydrological conditions and a change in methodology in counting cells across the two surveys (Materials and Methods). Independently from the methodological bias, the upstream-to-downstream increase in cell concentrations, shown as an increase in cell concentrations with increasing WTT on Fig. 1A, is most likely the result of high nutrient availability (Fig. S5) and aligns well with another previous investigation along the Danube River [21], as well as with reports from other river systems such as the Australian Murray River [22] and the river Rhine [23].

**Table 1:** Summary table of descriptive statistics on measured and calculated parameters for both surveys. *n*(JDS2) = 71–75, *n*(JDS3) = 54.

	Cumulative travel time, *tt*_*cum*_	*TCC*	*BSP*	*BSPc*	*CD*_*d*_	*CD*	a*CP*_*h*_	*CP*_*tot*_
	[days]	[10^9^ cells l^−1^]	[μgC l^−1^ h^−1^]	[fgC cell^−1^ h^−1^]	[day^−1^]	[cell divisions/~ 2600 km]	[10^6^ cells l^−1^ h^−1^]	10^9^ cells l^−1^
	Calculated	Measured	Measured	Calculated	Calculated	Calculated	Calculated	Calculated
This study								
JDS2 median (range)	33.7	1.71 (0.72–5.10)	0.32 *(*0.14–1.46)	0.19 (0.04–0.82)	0.12 (0.03–0.92)	4.2	8.65 (4.31–50.9)	9.21
JDS3 median (range)	49.7	13.9 (2.98–26.2)	1.15 *(*0.07–3.57)	0.09 (0.004–0.32)	0.12 (0.009–0.47)	6	75.7 (6.14–245.3)	95.9
Other studies								
Danube River, range [21]			0.95–4.83		0.09–1.04			
Danube River, range [33]			1.97–3.23					
Mississippi River, range [56]			0.7–81					

TCC, total cell count; BSP, bacterial secondary production; BSPc, bacterial secondary production per cell; CD, cell division rate; aCP, absolute cell production.

**Table 2:** Summary table of linear models relating bacterial parameters (dependent variable) to water travel time (independent variable) for both surveys. The probability of an error by rejecting the null hypothesis of no change over time is <0.05 in all models listed, except for the “Simpson diversity (between consecutive sites)” model for JDS3 where it is >0.05.

	*TCC* change		*BSP* change		*BSP*_c_ change		Observed ASVs (alpha diversity)		Simpson diversity (between consecutive sites)		Nestedness (between consecutive sites)		Simpson diversity (among all sites)	
	Cells l^−1^ h_travel_^−1^		ngC l^−1^ h^−1^ h_travel_^−1^		10^−21^ g C cell^−1^ h^−1^ h_travel_^1^		ASV h^−1^		10^−4^ h^−1^		10^−4^ h^−1^		10^−4^ h^−1^	
	Slope ± std. error	Adj. *R* ^2^ (*n*)	Slope ± std. error	Adj. *R* ^2^ (*n*)	Slope ± std. error	Adj. *R* ^2^ (*n*)	Slope ± std. Error	Adj. *R* ^2^ (*n*)	Slope ± std. Error	Adj. *R* ^2^ (*n*)	Slope ± std. Error	Adj. *R* ^2^ (*n*)	Slope ± std. Error	Adj. *R* ^2^ (*n*)
**JDS2 bulk**	0.93 ± 0.35 × 10^6^	0.08 (71)	−0.57 ± 0.14	0.19 (71)	−422 ± 94	0.21 (71)								
**JDS2-FL**							−0.145 ± 0.038	0.28 (39)	−1.14 ± 0.55	0.08 (39)	0.27 ± 0.69	−0.02 (39)	4.84 ± 0.21	0.4 (820)
**JDS2-PA**							−0.181 ± 0.063	0.14 (47)	−1.86 ± 0.66	0.12 (47)	1.57 ± 0.74	0.07 (47)	3.17 ± 0.21	0.15 (1378)
**JDS3**	9.52 ± 1.59 × 10^6^	0.4 (54)	−0.79 ± 0.23	0.18 (52)	−119 ± 20	0.41 (52)	−0.063 ± 0.015	0.25 (54)	−2.31 ± 0.26	−0.004 (53)	−0.06 ± 0.20	−0.02 (53)	4.59 ± 0.07	0.74 (1431)

FL, free-living fraction (0.2–3.0 μm) of JDS2 samples; PA, particle-associated (>3.0 μm) fraction of JDS2 samples.

Results from correlation analyses and backward selection regression models suggest that the outliers to the overall trend in TCC occurring downstream of tributaries and large cities are associated with dynamics in nitrate, pH, conductivity, and total suspended solids. Compared to the regression models with WTT as a single independent variable, the inclusion of these water quality parameters improved the explained variability from 8% and 40% to 36% and 60%, for JDS2 and JDS3, respectively (simple linear regression models: Table 2 and Fig. 1A; multiple linear regression models: Table S1, Fig. S8).

### Trends in bacterial secondary production

BSP, the rate of biomass production for the bulk prokaryotic community, fell within the typical range for the Danube and other ecosystems (Table 1). BSP ranged from 0.14 to 1.46 μgC l^−1^ h^−1^ with a median of 0.32 μgC l^−1^ h^−1^ during JDS2 [20]) and from 0.07 to 3.57 μgC l^−1^ h^−1^ with a median of 1.15 μgC l^−1^ h^−1^ during JDS3 (Table 1, Fig. 2B). These rates formed the basis for subsequent calculations of doubling times and cell division rates (*CD_d_*). Linear models indicate a significant overall longitudinal decrease in *BSP* along the river, despite pronounced local increases observed in river sections with strong anthropogenic influence, downstream of the major cities (Table 2, Fig. 2B). Incorporating parameters associated with wastewater input and eutrophication, such as the fecal indicator *E. coli*, nitrate, and chlorophyll-a, improved the explained variability in the regression models from 19% and 18% (WTT alone) to 63% and 61% for JDS2 and JDS3, respectively (Table S1, Fig. S8).

Cell-specific bacterial secondary production (*BSP* per cell; *BSP*_c_) showed a pattern similar to bulk BSP, exhibiting a significant overall decrease over the longitudinal river transect (Fig. 2C, Tables 1 and 2). This decline corresponds to carbon incorporation deceleration of 21 000 and 600 carbon atoms per cell per hour^2^, for JDS2 and JDS3, respectively.

### Cell doubling times in the context of species sorting

Based on cell-specific BSP rates and mean cell biomasses (mBM_c_) calculated from cell volumes, we estimated the average cell doubling time for each site (Fig. S2A). The mBM_c_ ranged from 15 × 10^−15^ to 55 × 10^−15^ gC cell^−1^ for JDS2 and 6 × 10^−15^ to 36 × 10^−15^ gC cell^−1^ for JDS3. Median cell doubling times were 8.05 (JDS2) and 8.23 (JDS3) days, corresponding to median cell division rates (CD_d_) of 0.124 and 0.121, respectively (Fig. 2C, Table 1). These values are consistent with historical reports for the Danube [21] (Table 1). Similar to *BSP* and *BSP_c_*, the highest cell division rates were observed between Budapest (rkm 1630) and Belgrade (rkm 1159; Fig. 2C). Based on the median cell division rate for the bulk communities, a cell experiences 4.2 and 6.5 cell divisions (i.e. doubling events) during its ~33.7 and ~ 53.6-day-long journey along the ~2600 km longitudinal river section for JDS2 and JDS3, respectively. These cell division rates provide sufficient doublings for species sorting to act on the bacterioplankton community composition.

The calculated doubling times, in the context of WTT, are critical quantitative measures for understanding ecological processes. WTT is therefore the most applicable predictor when discussing the development of both native and allochthonous bacterial populations exposed to varying riverine conditions. As WTT is a function of flow velocity, its variability—driven by discharge dynamics and anthropogenic impoundments—can be assumed to directly impact the ecological processes observed.

### Total cell production along the river

We estimated the absolute cell production per travel time and liter (a*CP_h_*) between consecutive sites using the mean bulk BSP (m*BSP*_up⟷down_), the mean cell biomass at the upstream site (m*BM*_c up_), and WTT (tt*_up⟷down_*) (Fig. S6, SI: Formula Collection). The absolute cell production ranged from 4.3 × 10^6^ to 50.9 × 10^6^ cells l^−1^ h^−1^ during JDS2 and from 6.1 × 10^6^ to 245.3 × 10^6^ cells l^−1^ h^−1^ during JDS3, averaging 8.75 times higher during JDS3.

Summing these section-specific values yielded the total cell production in 1 l of water traveling the entire river (*CP_tot_*) as 9.2 × 10^9^ (JDS2) and 95.9 × 10^9^ cells (JDS3). Relative to the median standing stocks (median TCC: 7 × 10^9^ cells l^−1^ for JDS2 and 13.9 × 10^9^ cells l^−1^ for JDS3), the total cell production equals ~5.4 (JDS2) and 6.9 (JDS3) times the median standing stock. Because the observed TCC only increased approximately two to three times along the river (Fig. 1A), the excess produced cells must have been lost due to die-off, sedimentation, and predation. Given that allochthonous inputs (e.g. WWTPs and tributaries) are net sources of cells and were not accounted for in this calculation, the real cell loss rates along the river are likely even higher. These substantial cell replacement rates provide the basis for the high bacterial community turnover rates observed.

### Diversity and community turnover

**Figure 3:**
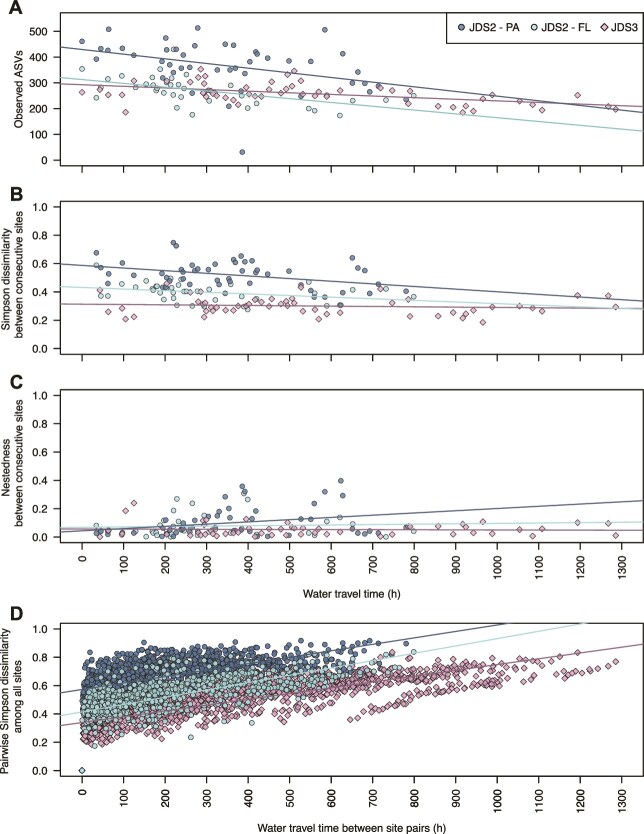
The gradual development of alpha and beta diversity with increasing water travel time (*tt_cum_*) during JDS3 (total community) and for the two separated size fractions studied during JDS2, representing free-living (FL) and particle-associated (PA) bacterioplankton. (A) Alpha diversity (observed ASVs). (B) Phylotype turnover (phylotype replacement, given as Simpson dissimilarity). (C) Nestedness component of beta diversity between two consecutive sites. (D) Pairwise phylotype turnover component among all sites. The colored lines represent fitted linear models for the respective datasets. Regression statistics and sample numbers are shown in Table 2.

The 16S rRNA gene amplicon sequencing data, available for the FL (0.2–3.0 μm) and PA (>3.0 μm) fractions of JDS2 and the bulk JDS3 samples, showed highly corresponding trends in bacterial alpha (species/phylotype richness within a sample) and beta diversity (differences in species/phylotype composition among samples) along the entire river (Fig. 3A and B, Fig. S7). Environmental analyses consistently indicated hydrological parameters (i.e. WTT) as the most important shaping factors for both alpha and beta diversity (Tables 2, S1, and  S2). Consequently, estimates of diversity changes were modeled as a function of WTT to account for the crucial role of bacterial generation time in species sorting.

Alpha diversity (count of observed ASVs) showed a gradual decrease along the river in both the pioneering JDS2 study [7] and in JDS3 (Fig. 3A, Table 2). The WTT-based simple linear regression model for alpha diversity revealed a decrease (phylotype loss) of 0.15 ± 0.04 amplicon sequence variants per hour [ASVs h^−1^] in the FL community and 0.18 ± 0.06 ASVs h^−1^ in the PA community during JDS2. During JDS3, the alpha diversity decreased by 0.06 ± 0.02 ASVs h^−1^ (Fig. 3A, Table 2). Correlation and stepwise multiple linear regression analyses consistently highlighted WTT as a significant factor influencing alpha diversity, often alongside various environmental parameters (Fig. S8, Table S1).

Beta-diversity, visualized by the nMDS of the Bray–Curtis dissimilarity matrix, confirmed the gradual development of the bacterial communities in all three datasets (Fig. S8). Variation in community composition was best explained by geomorphological and hydrological measures such as “distance to river mouth” (river kilometer), “median dendritic stream length,” catchment area, and WTT, with coefficients of determination as high as *R*^2^ = 0.91 (river km, JDS3, and WTT, JDS3). Associations with physicochemical parameters, nutrients, chlorophyll-a, and the fecal indicator *E. coli* had lower coefficients (Table S2).

We partitioned beta diversity, often estimated by Bray–Curtis dissimilarity, into ‘nestedness’ and ‘turnover’ on the basic mechanisms driving differences in community heterogeneity among sites: the loss of phylotypes and their replacement, respectively [47, 56]. Nestedness was generally minor, ranging from 0.001 to 0.397 with a median of 0.045 (FL JDS2), 0.055 (PA JDS2), and 0.036 (JDS3) between consecutive sites along the river continuum (Fig. 3C). In contrast, turnover dominated, varying between 0.18 and 0.75 between successive sites along the river continuum, with an overall trend of lower turnover towards the river mouth (Fig. 3B, Table 2). Turnover changed by a factor of 0.426 ± 0.017 (JDS2-FL), 0.250 ± 0.015 (JDS2-PA), and 0.592 ± 0.01 (JDS3) over the entire sampled continental river transect. Along the entire river, turnover was estimated to be between 0.92 and 0.96, contributing 94%–97% to beta diversity changes. This dominance of replacement over loss was further supported by a persistence analysis of headwater-associated phylotypes. Only a small fraction of ASVs detected in the three uppermost sites persisted throughout the river continuum, with 7% (JDS2-FL), 3% (JDS2-PA), and 11% (JDS3) remaining present in ≥90% of downstream samples. Hence beta-diversity patterns in the Danube River mainly resulted from phylotype replacement processes rather than the progressive loss of a conserved core community. This high turnover rate (estimates close to one) emphasizes an almost complete exchange of phylotypes throughout the continental river system under low base flow conditions, consistent with the previous observation of an increase of freshwater- and lake-bacteria downstream [7].

### Growth patterns of individual taxa and the increase of small cells

**Figure 4:**
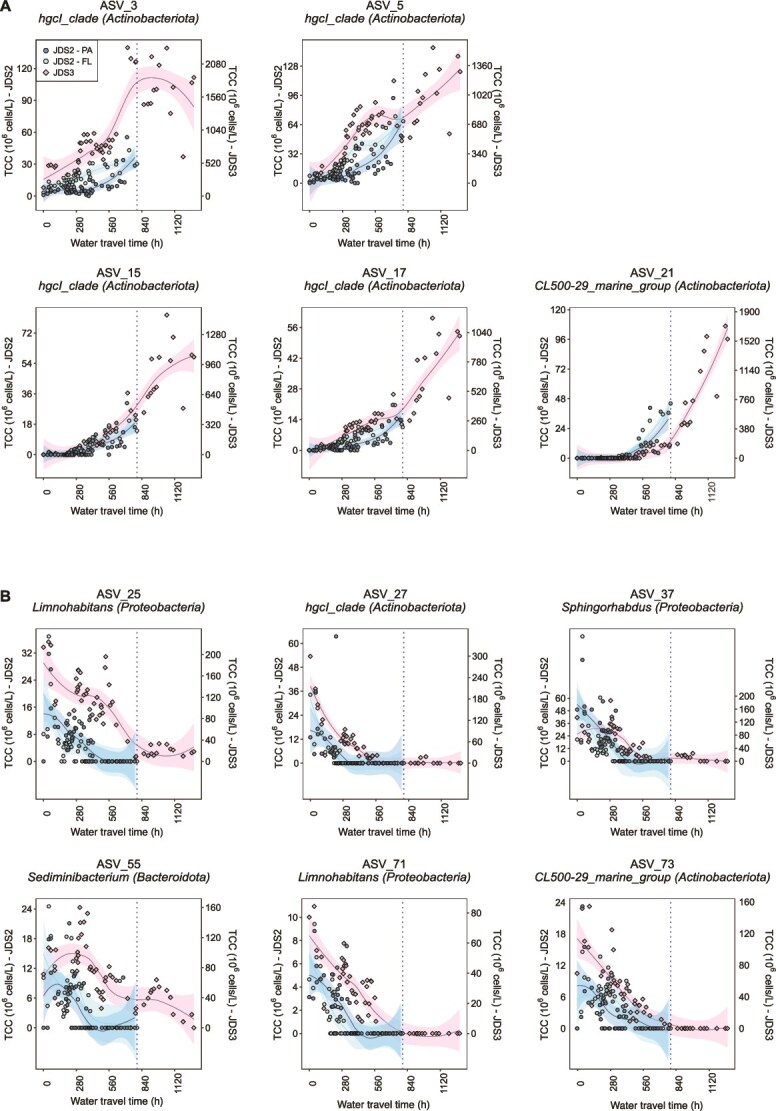
Changes in the abundance of selected phylotypes during their travel along the Danube River. (A) Phylotypes with increasing abundance. (B) Phylotypes with decreasing abundance. The second line of the graph titles gives the assigned genus (phylum). The lines show the fitted generalized additive models (R package *mgcv*), the ribbon marks their Bayesian credible interval (similar to a confidence interval). The dotted line marks the total travel time during JDS2. JDS2-FL, free-living fraction, JDS2; JDS2-PA, particle-associated fraction, JDS2.

To further resolve the re-occurring patterns between JDS2 and JDS3, growth patterns of individual taxa were estimated. Absolute phylotype abundances, estimated from relative abundances as retrieved from amplicon sequencing and the *TCC* for each sample, were fitted by generalized additive models. From the individual models, we extracted ASV growth characteristics. ASVs affiliated with the phylum *Actinobacteriota* showed particularly high rates, growing from zero or nearly zero up to 16% of the total community along the river, reaching an estimated absolute abundance of 2.3^*^10^8^ cells l^−1^ (ASV_3 during JDS3, Fig. 4, Table S3). Based on our other ASV growth estimate, site-to-site differences in absolute ASV abundance, we observed ASVs with cell division rates exceeding BSP-based bulk rates by 100 times over short stretches of the Danube River in both surveys (Table S3), though with great variability. The increase of competitive *Actinobacteria* coincided with or followed alterations in flow dynamics caused by, for example, damming at the Iron Gate hydropower plants, further confirming that local environmental shifts can play a major role in phylotype selection (Fig. 4) [37]. However, the observed net growth rates (sum of cell production and cell loss) are ~50–100 times lower than the *ex situ* growth rates for 20 different bacterial strains from a polluted river in Belgium [57]. This discrepancy highlights the role of cell losses, not investigated in the Belgian study but inherently incorporated in our observed growth patterns, and might also be due to differences in the bacterial strains and/or the nutrient profile of the river water.

Downstream decreases in abundance were observed for several other phylotypes, including *Limnohabitans* and the “CL500-29 marine group” (Fig. 4, Table S4). For example, ASV_27 peaked at 3^*^10^8^ cells l^−1^ upstream before decreasing to undetectable levels by the middle of the river. These declines reflect either lower competitiveness or higher specific loss rates. It further emphasizes that individual taxa within a community are continually subjected to asynchronous fluctuations in growth and death rates due to environmental stochasticity, resource competition, and predation. This high variability among individual taxa points to compensatory behavior: when one taxon increases dramatically, competing or functionally redundant species often experience a corresponding decrease. These fluctuations are expected to decrease due to a longitudinal decrease in BSP and are supported by a decrease in community turnover. However, physiological compensation through a shift toward smaller cell sizes, which require less biomass per division, ensures that the community’s total cell replacement capacity remains intact despite the macroscale BSP decline.

### Discontinuities in longitudinal trends

In addition to the modeled general trends along the longitudinal river transect, we also observed localized discontinuities. These were particularly evident for bacterial numbers (TCC), BSP, and their derivative parameters, such as carbon incorporation per cell, cell division rate, and cell production per kilometer, as well as for phylotype and community turnover rates (Fig. S6). For example, *BSP, BSPc*, and cell production peaked in the river section between the three large cities, Vienna (rkm 1919), Budapest (rkm 1660–1630), and downstream Belgrade (rkm 1159). These higher values were likely facilitated by increased organic matter and phytoplankton growth due to sewage input from the large cities and from intensive agriculture (as indicated by chlorophyll-a concentrations, [20] and Fig. S3). A correlation between *BSP* and phytoplankton growth was also observed during the first Joint Danube Survey conducted in 2001 [58]. The subsequent decrease in these parameters and in *TCC* downstream of Belgrade in the backwater of the first Iron Gate reservoir (rkm 1040) was accompanied by decreasing chlorophyll-a and suspended solid concentrations, alongside increasing zooplankton abundance (Rotifers, Cladocera, and Copepods; [59]). These shifts are all likely a consequence of large-scale damming at the Iron Gates.

Backward selection revealed a good model for observed ASV counts with multiple regressions only in the case of JDS3 and only when WTT was included, alongside environmental factors (pH, conductivity, organic matter, and nutrients), whereas variations in BSP, TCC, and observed ASVs are predictable by a combination of hydrological and water quality parameters, beta-diversity patterns follow a gradual development throughout the river. This development is best predicted by WTT, whereas environmental parameters showed low correspondence with this general trend. Consequently, all models predict a time-dependent evolution of the community during its travel (see also nMDS plot, Fig. S8) down the continental river. WTT thus can be argued to provide the best-suited parameter for predicting continental trends, and the inclusion of environmental factors serves to fine-tune these overall trends and pinpoint to local discontinuities.

### Temporal monitoring reveals higher alpha diversity with increased discharge

**Figure 5:**
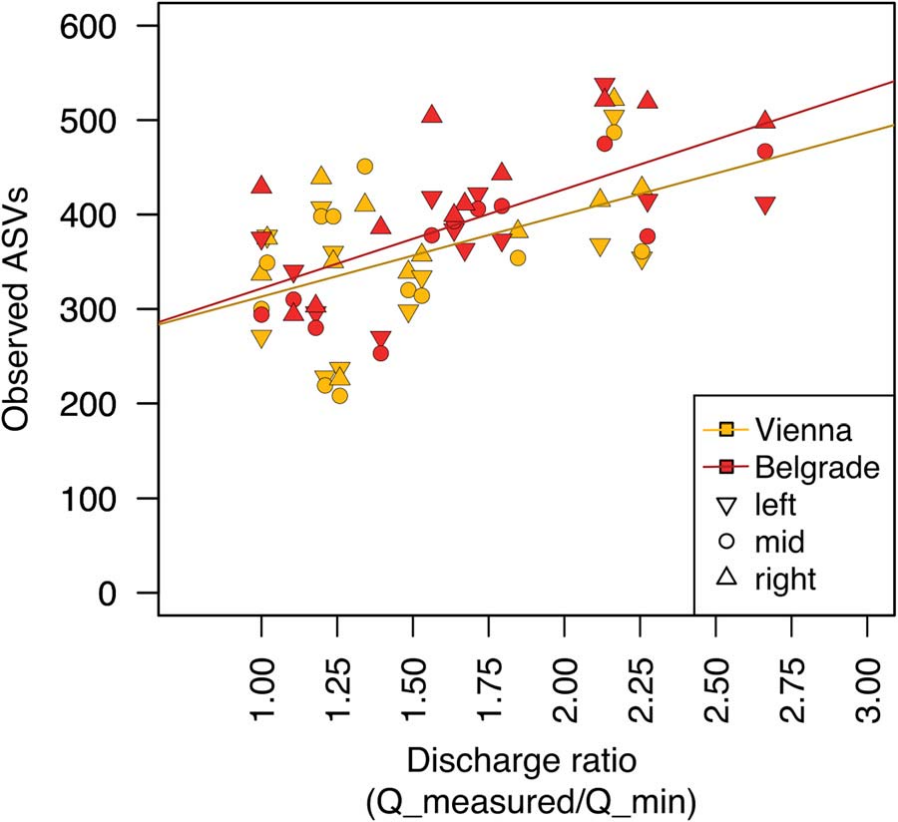
Correlation between alpha diversity (observed ASVs) and prevailing discharge levels during the 15-month monitoring of bacterial community dynamics at two large cities along the Danube River from January 2014 to January 2015 in Vienna [AT], rkm 1919 and Belgrade [RS], rkm 1159. The *x*-axis depicts the ratio between the prevailing discharge at the respective sampling date normalized to the minimum discharge measured at the respective site during the entire monitoring. Left, mid, and right refer to the sampling point along the river transect (close to the left shore, mid-stream, and close to the right shore). The lines represent the linear models relating the discharge ratio to observed ASVs for Vienna and Belgrade (see model details in the text).

In addition to the two longitudinal surveys, we conducted a monthly monitoring at the major cities Vienna and Belgrade over a 1-year period. This yearly cycle revealed a positive response of alpha diversity (observed ASVs) to increasing discharge. To allow for comparison between the two sites, which have distinct discharge regimes, we normalized the discharge measured at each sampling date to the minimum discharge observed at the respective location (defined as the “discharge ratio”). The resulting linear models of discharge-dependent response in observed ASVs for the two monitoring sites predict that a doubling in discharge results in an increase in richness of 87 ± 28 ASVs at Vienna (estimate ± std. error, *P_slope_* = .003, adjusted *R*^2^ = 0.21) and 105 ± 20 ASVs at Belgrade (*P_slope_* < .001, adjusted *R*^2^ = 0.44, Fig. 5).

The generalization of this linear model estimate to the entire Danube River or other continental drainage systems requires further investigation. Such future studies should address additional factors that explain the observed large residuals. High-resolution data on the hydrological history (i.e. whether sampling occurred at the beginning or at the end of a flood event), which likely affect the extent of cell mobilization from the riparian zone [30, 31, 60], could explain parts of the residuals in alpha diversity. Nonetheless, our finding is supported by previous observations that local increases in discharge, typically from snowmelt or precipitation events within a subcatchment, enhance species richness due to greater groundwater input and surface runoff [9, 31, 60].

### Conclusions

The essential role of the hydrogeological structure in lotic aquatic systems—representing the link between land and the ocean in the global water cycle—in controlling macroecological processes has been widely suggested and debated in numerous studies [7–12]. Here, we report reproducible patterns in BSP, as well as cell, phylotype, and community turnover rates along the large Danube River. This allowed us to develop a predictive framework for bacterial diversity and function along a continental drainage system. Our model estimates allowed for general predictions including increasing cell numbers, decreasing secondary production, and decreasing alpha diversity (phylotype richness) with increasing WTT. This trend, combined with observations of decreasing cell volumes [7, 20], points to major bacterial physiological responses, specifically a shift toward streamlined and less active cells, ultimately driving major ecosystem-scale functional responses (e.g. BSP). In addition to these overall trends, we observed local discontinuities in diversity and function. These breaks were possibly associated with locally intensive agriculture and wastewater input from municipalities, indicating that major anthropogenic modification can disrupt general macroecological patterns. Beyond these qualitative predictions, our models provide the first quantitative rate changes in macroecological patterns on a continental scale. Key quantitative findings are that hourly carbon incorporation per cell decreased by 6000 to 21 000 atoms every hour travelled, that *BSP* decreased by 0.43–1.02 ngC l^−1^ h^−2^, that a doubling of discharge results in an increase in richness of around 87–105 ASVs at a single site, and that phylotype turnover along the entire river was between 0.92 and 0.96, dominated by phylotype replacement resulting in an almost complete exchange of phylotypes throughout the continental river system under base flow conditions. Although these estimates are based on two consecutive surveys in a single system, and their immediate generality can be challenged, our study provides a framework for predicting macroecological change in microbial diversity and functions affected by human impacts. For instance, climate warming resulting in decreased flow velocities associated with less streamflow in Eastern Europe [61] or the construction of dams can be predicted, based on our data, to cause a measurable decrease in alpha diversity and an increase in community change. Consequently, the bacterial diversity reaching the Danube River’s estuary and the Black Sea will decrease, as previously proposed for another river system [62]. Furthermore, our findings demonstrate physiological shifts toward smaller, more streamlined cells with lower per-cell activity reaching the sea. This physiological change, combined with the overall decrease in BSP despite increased total cell numbers, reveals major functional changes underlying carbon cycling along the continental river system. Altogether, our findings highlight the central importance of hydrological parameters such as discharge and WTT (or WRT) in determining bacterioplankton macroecology—from cell physiology and diversity to community function—along the hydrological path from land to sea.

## Supplementary Material

2_Supplementary_JDS2vs3_20260129_wrag013
