# Supplementary material for: Hydrological regime of a continental river system predicts bacterial macroecological patterns
Source: ISME J. 2026 Feb 2;19(1):wrag013. doi: 10.1093/ismejo/wrag013 (PMC12927878; doi:10.1093/ismejo/wrag013)
Supplement: 2_Supplementary_JDS2vs3_20260129_wrag013 [file 2_supplementary_jds2vs3_20260129_wrag013.docx]

**Supporting Information**

**Bacterial macroecological patterns in a continental river system are predictable from hydrological regime**

Katalin Demeter^1,2,3⊗^, Domenico Savio^1,2,3,4⊗^, Alexander K.T. Kirschner^2,4,5,*^, Georg H. Reischer^1,2,6^, Stoimir Kolarevic^7^, Juraj Parajka^8^, Julia Derx^2,8^, Stefan Jakwerth^2,5^, Christian Wurzbacher^9^ , Alfred P. Blaschke^2,8^, Robert L. Mach^10^, Günter Blöschl^8^, Andreas H. Farnleitner^1,2,4,*^, Alexander Eiler^11,12^

1 Research Group Environmental Microbiology and Molecular Ecology, Institute of Chemical, Environmental and Bioscience Engineering, TU Wien, Vienna, Austria

2 Interuniversity Cooperation Centre Water and Health, www.waterandhealth.at

3 Centre for Water Resource Systems (CWRS), TU Wien, Vienna, Austria

4 Karl Landsteiner University of Health Sciences, Division Water Quality and Health, Krems an der Donau, Austria

5 Institute for Hygiene and Applied Immunology, Water Microbiology, Medical University Vienna, Vienna, Austria

6 TU Wien, IFA-Tulln, Austria

7 University of Belgrade, Serbia

8 Institute of Hydraulic Engineering and Water Resource Management, TU Wien, Vienna, Austria

9 Chair of Urban Water Systems Engineering, TUM, Munich, Germany

10 Institute of Chemical, Environmental and Bioscience Engineering, TU Wien, Vienna, Austria

11 Department of Biosciences, Centre of Biogeochemistry in the Anthropocene, Section for Aquatic Biology and Toxicology University of Oslo, Oslo, Norway

12 eDNA solutions AB, Mölndal, Sweden

* Corresponding author: Alexander K.T. Kirschner, Andreas H. Farnleitner

**Email: [alexander.kirschner@meduniwien.ac.at](mailto:alexander.kirschner@meduniwien.ac.at), [andreas.farnleitner@tuwien.ac.at](mailto:andreas.farnleitner@tuwien.ac.at)**

**This document includes:**

Supplementary Materials and Methods

Formula Collection

Figures S1 to S5

Tables S1 to S2

SI References

## Supplementary Materials and Methods

## Sample collection, DNA extraction and 16S rRNA gene amplicon library preparation

Sample collection and further experimental procedures of Joint Danube Survey 2 for results that were re-analysed in the current study were published by Savio, et al. ^7^.

During Joint Danube Survey 3, duplicate samples (biological replicates) were collected from midstream in sterile 1 L glass flasks from a water depth of approximately 30 cm for DNA analysis. Glass flasks were sterilized by rinsing with 0.5% HNO_3_ and autoclaving. Depending on the expected biomass concentration, 120-300 mL of river water were filtered through 0.2 µm pore-size polycarbonate filters (Cyclopore, Whatman, Germany) by vacuum filtration for biomass concentration. The filters were stored at -80 °C until DNA extraction.

Genomic DNA from JDS3 samples was extracted as for JDS2 using a slightly modified protocol of a previously published phenol-chloroform and bead beating-based procedure ^45^ using isopropanol instead of polyethylene glycol for DNA precipitation. Total DNA concentration was assessed applying the Quant-iT PicoGreen dsDNA Assay Kit (Life Technologies Corporation, USA). 16S rRNA gene concentrations in the DNA extracts were quantified using domain-specific quantitative PCR as described previously ^7^. DNA extracts were normalized with regard to 16S rRNA gene concentrations in order to use standardized numbers of bacterial 16S rRNA gene templates for amplification and barcoding in a two-step barcoding procedure. In short, the first-step primers (341f and 805r) contained adapters for introducing Illumina adapters, and dual barcodes were used in the second step. The first-step PCR primers were thus (adapter sequence, followed by primer sequence) adapter-341f (‘5 - ACACTCTTTCCCTACACGACGCTCTTCCGATCTNNNNCCTACGGGNGGCWGCAG-3’) and adapter-805r (‘5-AGACGTGTGCTCTTCCGATCTGACTACHVGGGTATCTAATCC-3’). The first-step amplicon PCR (ampPCR) was carried out in duplicate in 20 µL reaction mixtures containing 1 × Q5 reaction buffer, 0.2 mM dinucleoside triphosphates (dNTPs), 0.5 µmol L^-1^ forward and reverse primers, 0.4 U of Q5 high-fidelity DNA polymerase (New England BioLabs), as well as environmental DNA as template that was normalized to equal amounts of 16S rRNA gene copies prior to barcoding in order to increase comparability and reduce PCR bias. For normalization purposes, 16S rRNA gene copy concentrations in DNA extracts were determined by quantitative PCR as previously described ^7^. Cycling conditions for 1st-step ampPCR were 98 °C for 1 min, followed by 20 cycles of 98 °C for 10 s, 62 °C for 30 s, 72 °C for 30 s, and a final extension at 72 °C for 2 min. The duplicate products were pooled and purified using the Agencourt AMPure XP purification system (Beckman Coulter). The second PCR step containing variable combinations of primers with different multiplex-identifiers for sample-specific “barcoding” (forward, AATGATACGGCGACCACCGAGATCTACAC-[index]-ACACTCTTTCCCTACACGACG; reverse, CAAGCAGAAGACGGCATACGAGAT-[index]-GTGACTGGAGTTCAGACGTGTGCTCTTCCGATCT) binding to the first-step adapters and incorporating Illumina adapters was carried out in single 20 µL reactions. Reactions contained 1 × Q5 reaction buffer, 0.2 mmol L^-1^ dinucleoside triphosphates (dNTPs), 0.25 µ mol L^-1^ forward and reverse index primers, 0.4 U of Q5 high-fidelity DNA polymerase (New England BioLabs) and 2 µL of purified amplicons from 1st-step ampPCR as template. Cycling conditions for the 2nd-step index PCR (idxPCR) were 98 °C for 1 min, followed by 15 cycles of 98 °C for 10 s, 66 °C for 30 s, 72 °C for 30 s, and a final extension at 72 °C for 2 min. PCR products (amplicon libraries) were purified as described above and quantified with the PicoGreen kit (Life Technologies). Products were sequenced (2 x 300 bp) at the SciLifeLab SNP/SEQ sequencing facility at Uppsala University, Uppsala, Sweden, on a MiSeq System (Illumina) in two runs. A subset of the JDS2 samples (n=66) were processed twice, as technical replicates. Technical replicates showed high similarity (Figure S1).

## Geomorphological measures and estimation of in-stream water residence time

**Geomorphological parameters** including “catchment area”, “mean dendritic stream length” and “cumulative dendritic distance upstream” were calculated for each sampling site as described previously ^7^. The “mean dendritic stream length” at a respective sampling site gives the average flow distance travelled by a drop of water through the entire catchment area to a particular sampling site (under the assumption that spring discharges are randomly distributed in the catchment), and therefore serves as a proxy for the average residence time in the stream (assuming constant flow velocities). In contrast, the “cumulative dendritic distance upstream” was calculated as the sum of all mapped flow paths to a respective sampling site and represents a geometric parameter indicative of the drainage density, but not of residence times ^7^.

In contrast, **in-stream water residence time (“travel time”)** along the main stream of the Danube River (*i.e.,* without tributaries) from the very upstream site (Ulm, Germany) to the river mouth (Black Sea, Romania) was also estimated by an alternative approach based on on-site measurements of mean flow velocities at each sampling site. Here, **“cumulative travel times”** for JDS2 and JDS3 were calculated by cumulating the calculated flow times between each two consecutive sampling sites. These, in turn, were estimated on the basis of averaged on-site 3D flow velocity measurements from two consecutive sampling sites and the flow distance between them. These calculations resulted in estimated “cumulative travel times” of ~ 33.7 days and ~ 49.7 days from rkm 2600 to rkm 18 and rkm 2581 to rkm 18 for JDS2 and JDS3, respectively.

## Formula Collection

This section complements Figure S2.

1. **Doubling time and cell division rate (for each site)**

***Bacterial numbers and biometry***

Mean cell volumes per cell (m*V_c_*) [µm^3^ cell^-1^] were determined by averaging calculated cell volumes from at least 100 randomly selected cells per sample as determined by epifluorescence microscope-based morphometry by measuring the diameter (cocci) or length and widths (all other morphotypes) for each cell and calculating volumes either as spheres (for cocci), cylinders with two half spheres at the ends (rods, curved rods, filaments). Cell volumes for single cells were calculated as follows:

1. *V_rounded cylinder/sphere_* = (length - width/3) × (π/4 × width × width)
   *V_cocci_*  = *V_sphere_*

*V_rods, filaments_*  = *V_rounded cylinder_*

**Mean cell volumes** (m*V_c_*) [µm^3^ cell^-1^] for each site was calculated as the weigthed mean considering each cell type (cocci, rods, curved rods and filaments in JDS2; small and large cells in JDS3).

**Mean cell biomass** (m*BM_c_*) [fgC cell^-1^] was computed for each site by multiplication of calculated mean cell volume at the respective site (m*V_c_*; in µm^3^) with a commonly applied dry weight conversion formula of 120 × m*V_c_*^0.72^ - representing the functional allometric relationship between cell biomass (in femtograms C) and mean cell volume (in cubic micrometers; m*V_c_*) of bacteria ^65^.

1. *BM_c_* = 120 × m*V_c_*^0.72^

***Bacterial secondary production***

**Bacterial secondary production** (*BSP*) [µgC L^-1^ h^-1^] of the bulk prokaryotic communities was determined based on ^3^H-Leucine incorporation rates (LI) [mol L^−1^ h^−1^],

1. *BSP* = LI · 131.2 · (% Leu)^-1^ · (C/protein) · ID

where the constant of 131.2 represents the molar mass [g mol^-1^] of leucine, % Leu is the fraction of leucine in protein (0.073) ^66^, C/protein is the ratio of cellular carbon to protein (0.86), and ID is the isotope dilution. For ID, the value of 1 was used based on leucine uptake kinetics experiments conducted in parallel with sample measurements. These were conducted on representative samples from along the river in order to determine the concentration at which the uptake of ^3^H-Leucine was saturated, and no isotope dilution was observed ^20^. Based on these experiments, a final concentration of ^3^H-Leucin of 100 nmol L^-1^ was selected for both surveys.

**Bacterial secondary production per cell** (*BSP*_c_) [fgC h^-1^ cell^-1^] was calculated for each sampling site by dividing the total *BSP* [µgC L^-1^ h^-1^] by the total cells counts (*TCC*) [cells mL^-1^] as determined with epifluorescence microscopy.

1. *BSP*_c_ = *BSP* / (TCC × 10^3^) × 10^9^

***Doubling time and cell division rate***

**Doubling times** (*DT_d_,)* [days] were calculated by dividing the mean cell biomass (m*BM_c_*) [fgC cell^-1^] by the mean *BSP* per cell (*BSP_c_*) [fgC h^-1^ cell^-1^] at the respective site.

1. *DT_d_* = *mBM_c_* / *BSP_c_* × 24

**Daily cell division rates** (*CD_d_*) [day^-1^] for each sampling station were calculated as the reciprocal of daily doubling times (*DT_d_*).

1. *CD_d_* = *DT_d_* ^-1^
2. **Cell production rates (per travel distance or travel time)**

**Absolute cell production rates per hour** (a*CP_h_*) between two consecutive sites [cells mL^-1^ h^-1^] were calculated by dividing the mean *BSP* of the bulk bacterial communities of two sites (m*BSP*_up⟷down_) [µgC L^-1^ h^-1^] by the mean biomass per cell at the upstream site (m*BM_c up_*) [fgC cell^-1^]. To obtain cell production rates per mL and hour, m*BSP*_up⟷down_ had first to be multiplied by 10^9^ and then be divided by 10^3^ to obtain production in fg C per mL:

1. a*CP_h_* = (m*BSP_up⟷down_* × 10^9^ / 10^3^) / m*BM_c up_*

**NOTE**: In case no *BSP* measurements were available for neither site, the median *BSP* from all sites was used.

**Absolute cell production between two consecutive sites** (a*CP_up⟷down_*) [cells mL^-1^] was estimated by multiplication of a*CP_h_* with the calculated travel time in hours (*tt_up⟷down_*) [h] between two consecutive sites.

1. a*CP_up⟷down_* = a*CP_h_* × *tt_up⟷down_*

**Travel time between two consecutive** **sites** (*tt_up⟷down_*) in hours [h] was estimated by dividing the flow distance between two sites (*d_up⟷down_*) in km by the mean flow velocity (m*v_up⟷down_*) [m s^-1^] between the two sites as estimated by averaging the measured flow velocities at the two sites.

1. *tt_up⟷down_* = (*d*_up⟷down_ × 10^3^ / m*v_up⟷down_*) / 3600 s

Cumulative travel times in days [d] for the two surveys were estimated by simply summing up all between site travel times:

1. *tt*_cum_ = ∑ *tt_up⟷down_*

**Absolute cell production rate per km** (a*CP_km_*) between two consecutive sites [cells mL^-1^ km^-1^] was calculated by dividing the calculated absolute cell production between each two sites (a*CP_up⟷down_*) by the flow distance between two sites (*d_up⟷down_*).

1. a*CP_km_* = a*CP_up⟷down_* / *d_up⟷down_*

Estimated **total cell production along the entire river** (*CP_tot_*) [cells mL^-1^] during both surveys was calculated by summing up all calculated cell production values between all sites.

1. *CP_tot_* = ∑ *aCP_up⟷down_*

## Supplementary figures and tables


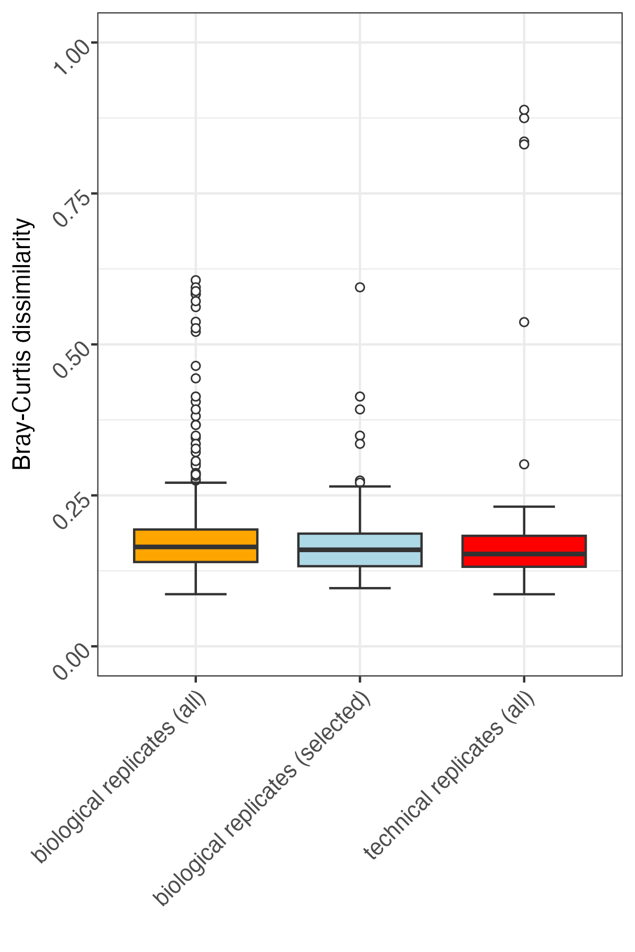


**Figure S1**. Quality control: bacterial community composition analysis of biological and technical replicates during JDS3. Biological replicates (all) cover all sites sampled (n_replicates_ = 658). Biological replicates (selected) are the sites presented in this paper, that is: mid-river samples from all main stem transects (n_replicates_ = 122) and the one-year long monitoring data from Vienna and Belgrade (n_replicates_ = 54). Technical replicates were prepared from a subset of the samples, therefore, these samples are presented with their biological replicate pair in the first and second pool, and with their technical replicate pair in the third pool.


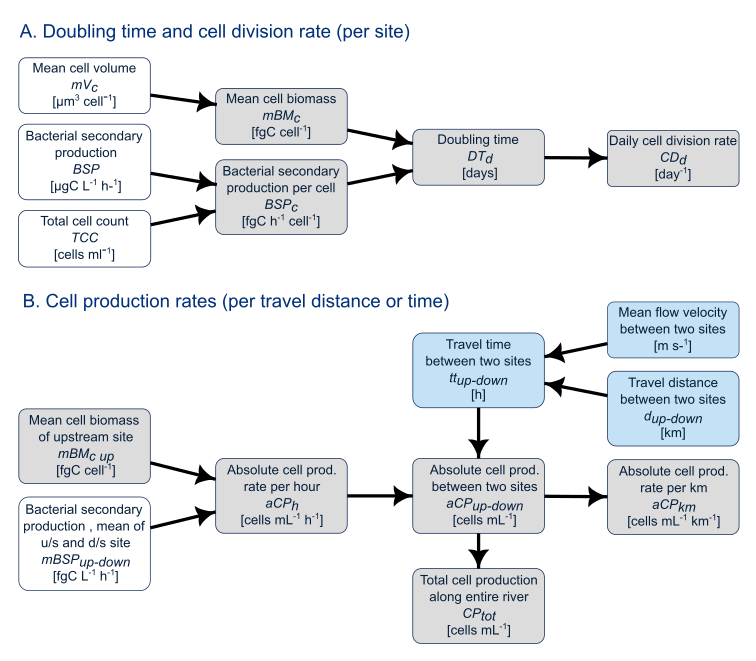


**Figure S2.** Schematic overview of calculation paths for bacterial cell division rates (**A**) and cell production rates (**B**). White background: measured microbiological parameters, grey background: calculated microbiological parameters, blue background: measured and calculated hydrological parameters. u/s: upstream, d/s: downstream.

**
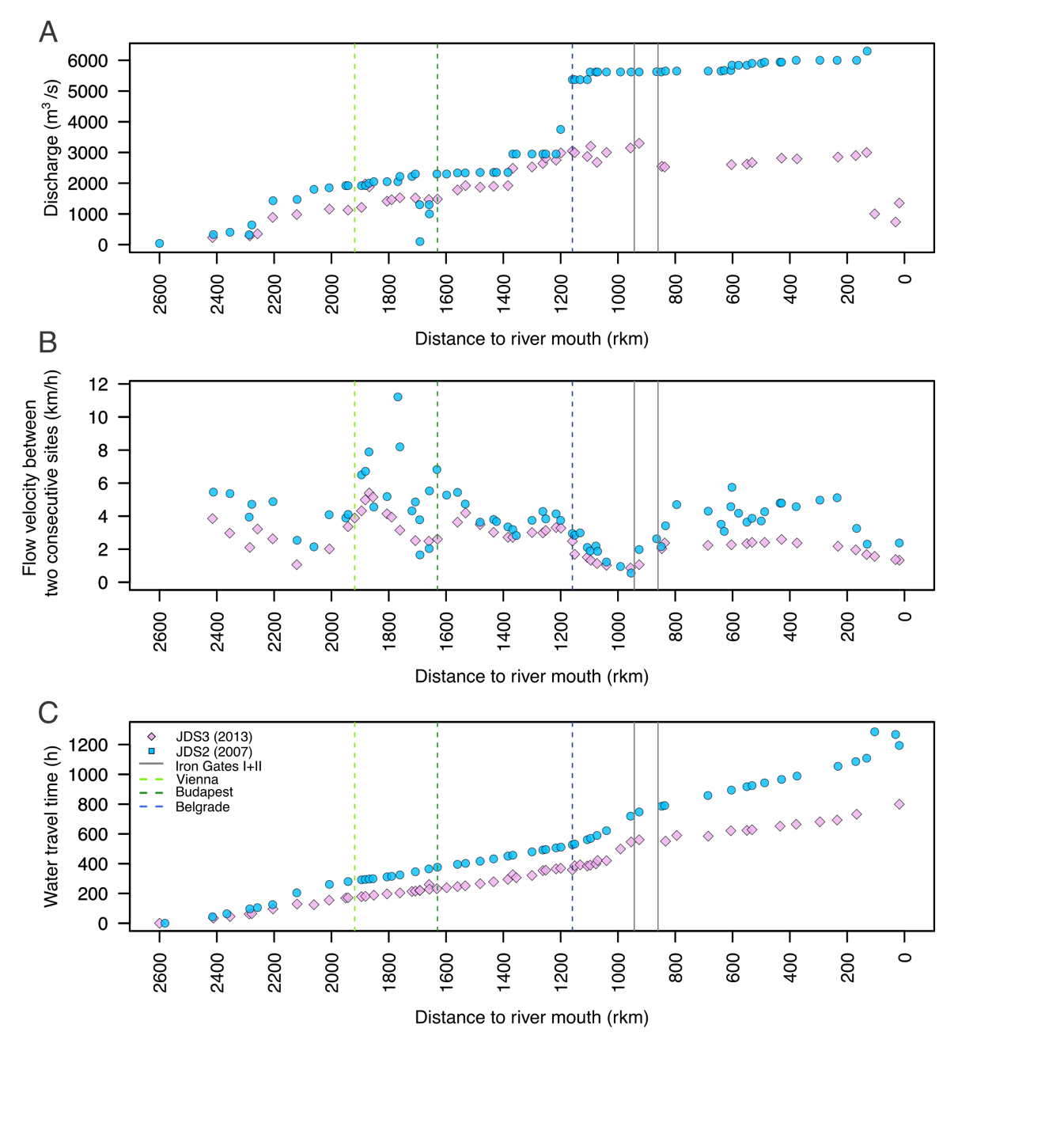
**

**Figure S3.** Hydrological parameters along the river Danube for both Joint Danube Surveys JDS2 and JDS3. **A** Discharge, **B** Flow velocity and **C** Water travel time. n(JDS2) = 32-75, n(JDS3) = 49-53.


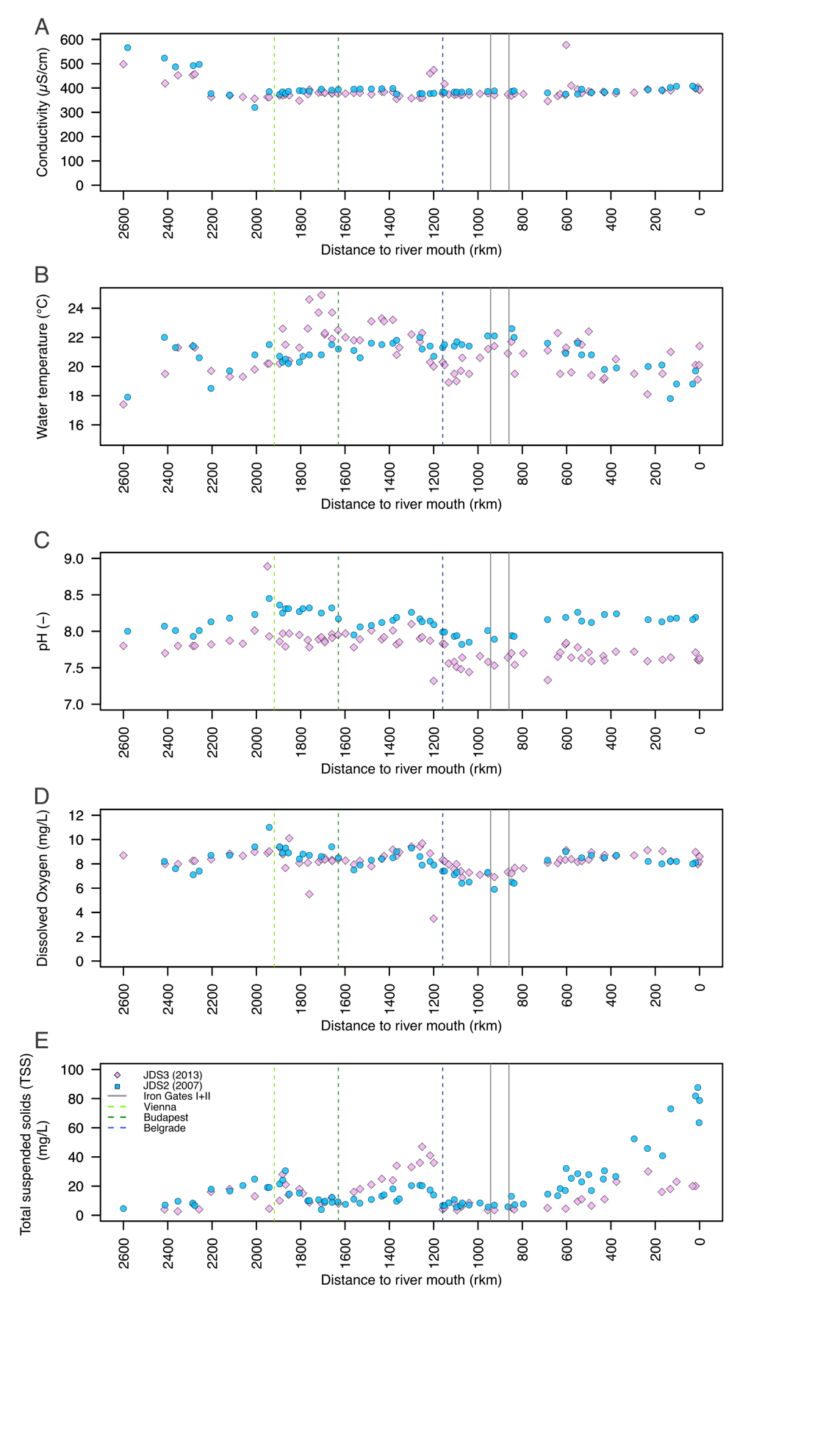


**Figure S4.** General water quality: physico-chemical parameters along the river Danube for both JDS2 and JDS3. n(JDS2) = 70-75, n(JDS3) = 51-54.


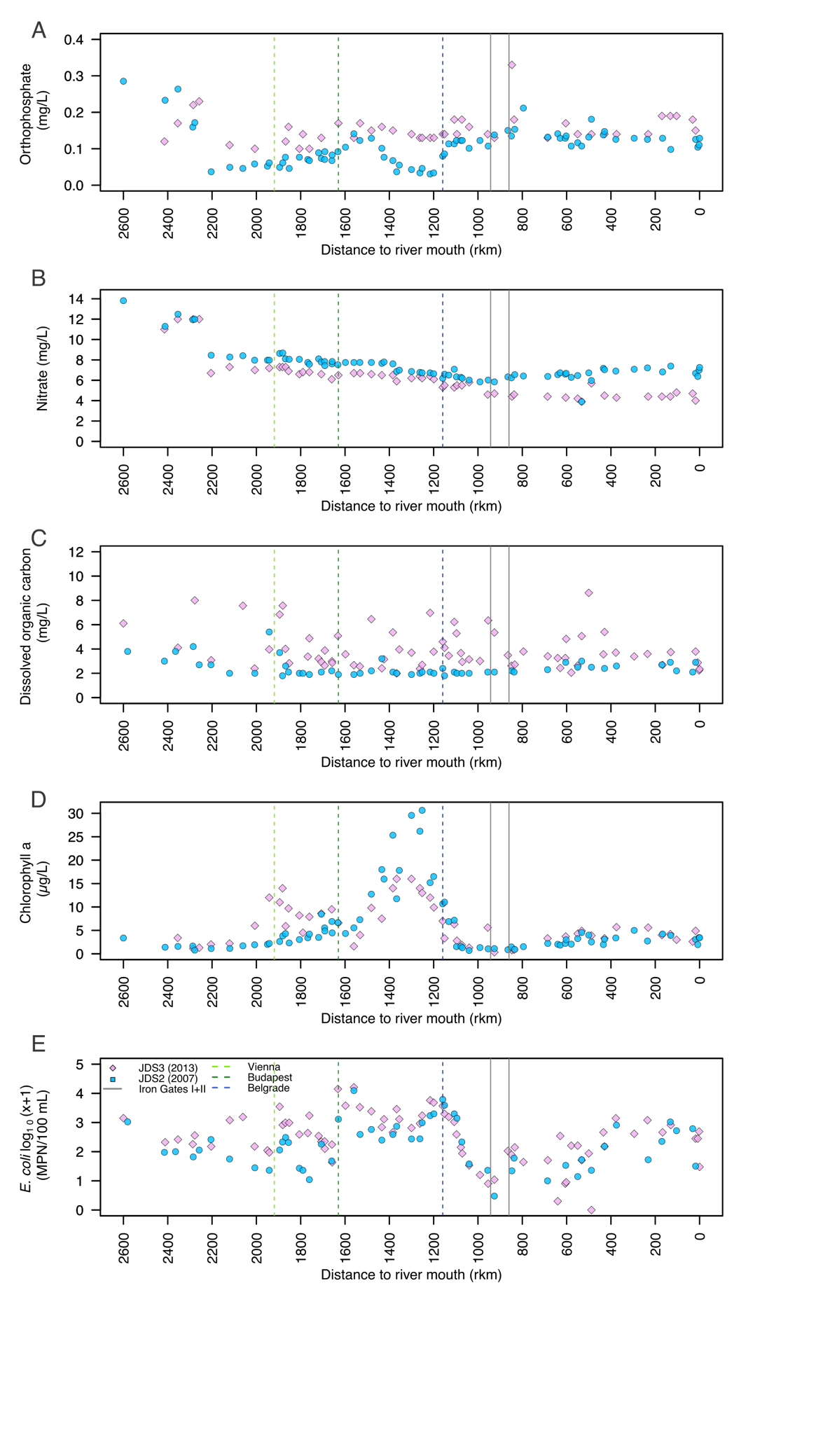


**Figure S5.** General water quality: nutrients, phytoplankton abundance indicator Chlorophyll-*a* and faecal indicator *E. coli* along the river Danube for both JDS2 and JDS3. n(JDS2) = 75, n(JDS3) = 49-53.


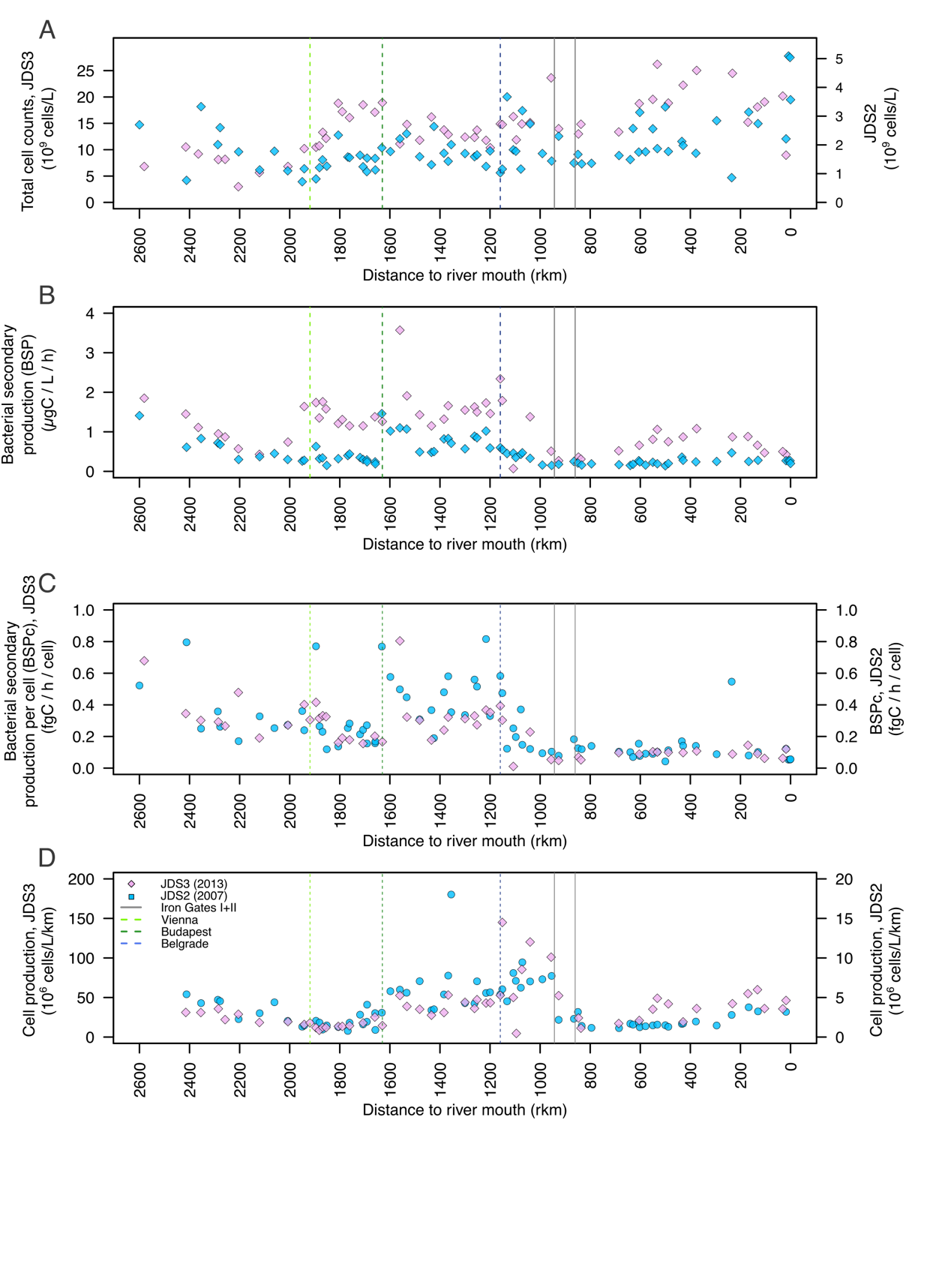


**Figure S6.** Trends in **A** Total cell counts (*TTC*), **B** Bacterial secondary production (*BSP*), **C** cell-specific bacterial secondary production rates (*BSP_c_*), **D** absolute cell production rates per km between two sites (a*CP_km_*) along the Danube River. n(JDS2) = 75, n(JDS3) = 54.


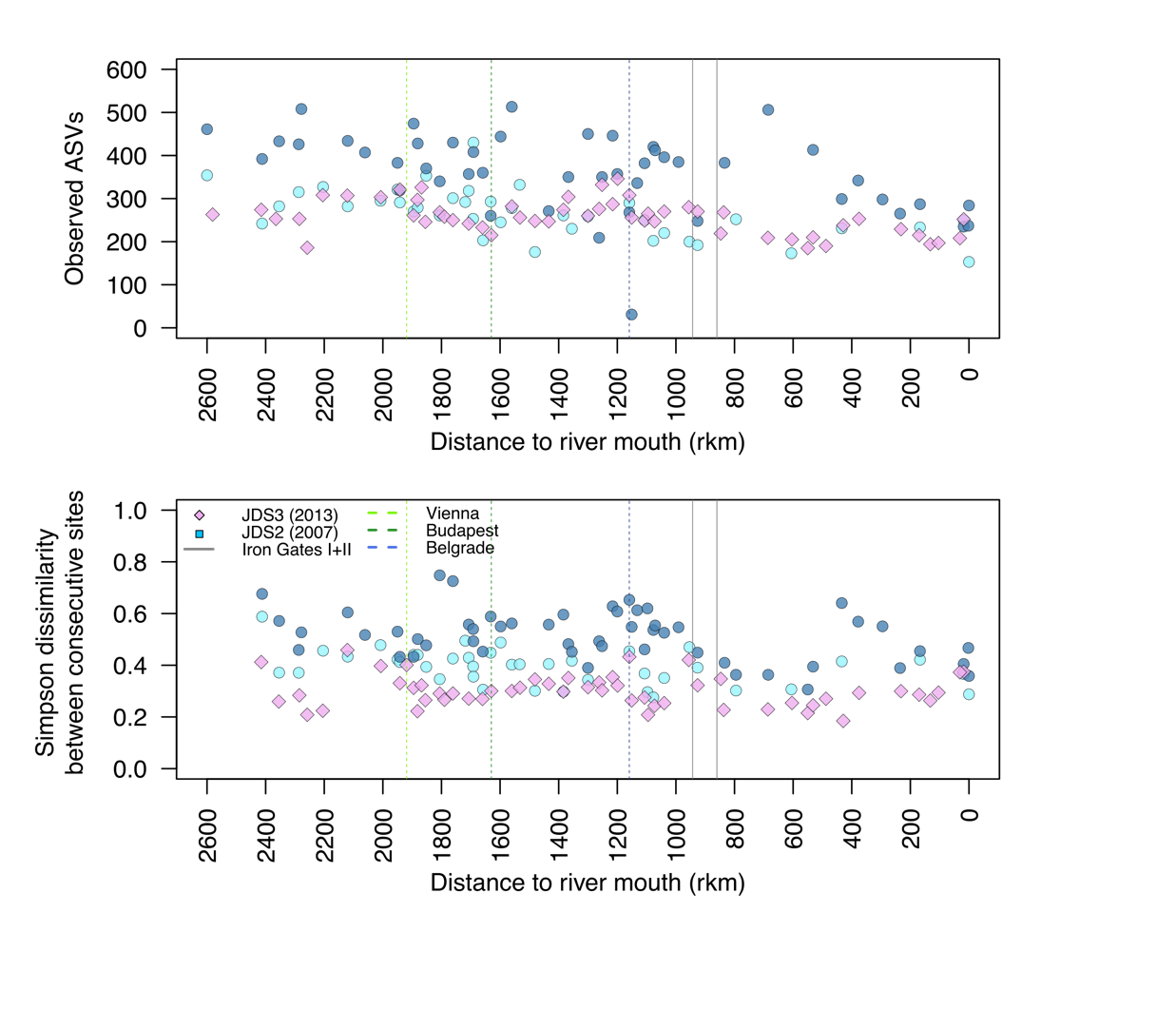


**Figure S7.** Trends in **A** alpha diversity (Observed ASVs), and **B** beta diversity (Simpson dissimilarity) between consecutive sites along the Danube River. n(JDS2) = 75, n(JDS3) = 54.


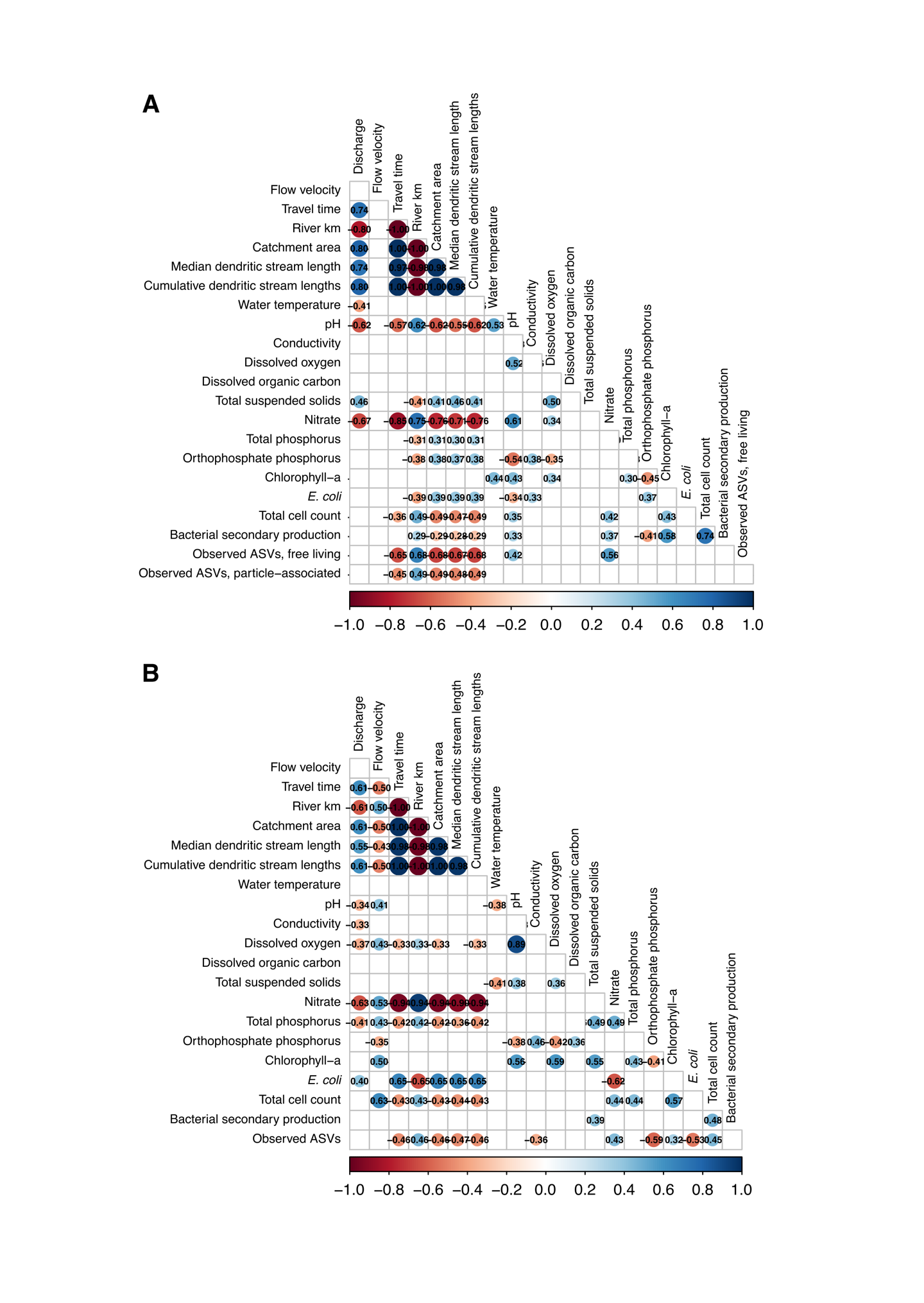


**Figure S7.** Spearman rank correlation matrix on pairwise complete cases of environmental variables as well as bacterial numbers, bacterial activity and community richness for **A** JDS2 (n = 16-75) and **B** JDS3 (n = 42-53). Only significant (*P* ≤ 0.05) correlations are shown. *P* values have been Benjamini-Hochberg-corrected.

Dendritic stream length refers to the measurement of the distance along the channel of streams within a dendritic drainage basin, a common drainage pattern that resembles tree branches. The median dendritic stream length is the median length for a given stream order. The cumulative dendritic stream length is the total length of all streams in the catchment.


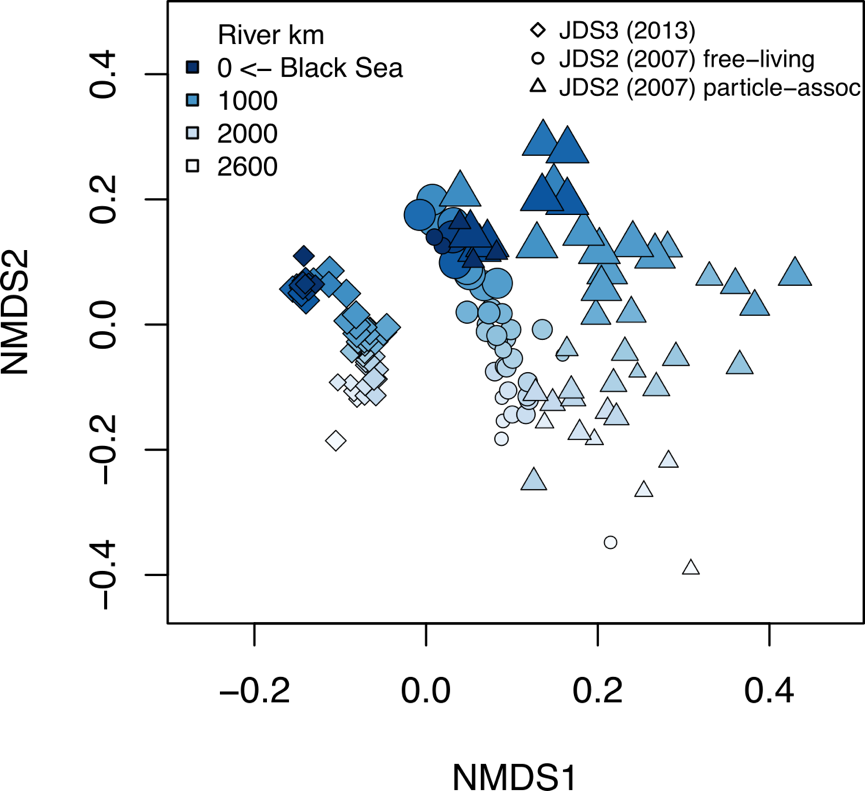


**Figure S8.** Non-metric multidimensional scaling (NMDS) of Bray-Curtis dissimilarities, illustrating the trends in longitudinal development of the bacterial communities along the river during the three studied datasets. The size of the symbols is proportional to the discharge at the respective site, the shade of blue indicates the location of the site along the river, from the most upstream site (white) towards the river mouth at the black see (rkm 0; blue).

**Table S1.** Multiple linear regression with backward selection between environmental variables and total cell counts, bacterial secondary production and Observed ASVs. The variables included in the starting set were: *Discharge, Flow velocity, Travel time, Water temperature, pH, Conductivity, Dissolved oxygen, Dissolved organic carbon, Total suspended solids, Nitrate, Total phosphorus, Chlorophyll-a, E coli, Total cell count, Bacterial secondary production, Observed ASVs*. Due to the high number of missing values, the following variables were excluded from the JDS2, JDS2-FL and JDS2-PA datasets: *Discharge, Flow velocity, Travel time, Observed ASVs* (except if Observed ASVs was the dependent variable of the model). Significance codes: *** *P* ≤ 0.001, ** *P* ≤ 0.01, * *P* ≤ 0.05. JDS2-FL: free-living fraction, JDS2; JDS2-PA: particle-associated fraction, JDS2.

| **Dataset** | **Model** | **Sample n** | **Variable** | **Slope** | **Adjusted *R*^2^** |
| --- | --- | --- | --- | --- | --- |
| JDS2 | Total cell count | 70 | pH | -1.30 x 10^6^* | 0.36*** |
|  |  |  | Conductivity | 4.88 x 10^3^ |  |
|  |  |  | Total suspended solids | 2.35 x 10^4^*** |  |
|  |  |  | Bacterial secondary production | 4.36 x 10^5^ |  |
| JDS3 | Total cell count | 43 | Discharge | 2.88 x 10^3^** | 0.60*** |
|  |  |  | pH | 2.25 x 10^7^*** |  |
|  |  |  | Conductivity | 1.37 x 10^5^*** |  |
|  |  |  | Dissolved organic carbon | -2.23 x 10^6^* |  |
|  |  |  | Total suspended solids | -1.26 x 10^5^* |  |
|  |  |  | Nitrate | -1.76 x 10^6^*** |  |
| JDS2 | Bacterial secondary production | 70 | Conductivity | 1.01 x 10^-3^ | 0.63*** |
|  |  |  | Dissolved organic carbon | 2.76 x 10^-3^ |  |
|  |  |  | Total suspended solids | -4.45 x 10^-3^** |  |
|  |  |  | Nitrate | 2.21 x 10^-1^** |  |
|  |  |  | Chlorophyll-a | 1.08 x 10^-2^** |  |
|  |  |  | Total cell count | 5.32 x 10^-8^ |  |
|  |  |  | *E. coli* | 1.94 x 10^-1^*** |  |
| JDS3 | Bacterial secondary production | 43 | Discharge | 1.26 x 10^-4^ | 0.61*** |
|  |  |  | Flow velocity | 7.80 x 10^-1^** |  |
|  |  |  | Water temperature | 2.53 x 10^-1^** |  |
|  |  |  | Dissolved oxygen | 2.46 x 10^-1^* |  |
|  |  |  | Total phosphorus | 10.7* |  |
|  |  |  | Chlorophyll-a | -6.48 x 10^-2^* |  |
|  |  |  | *E. coli* | 4.16 x 10^-1^*** |  |
|  |  |  | Observed ASVs | 2.84 x 10^-3^ |  |
| JDS2-FL | Observed ASVs | 34 | *E. coli* | 19.1 | 0.31** |
|  |  |  | Nitrate | 79.1** |  |
|  |  |  | Total cell count | -2.94 x 10^-5^* |  |
|  |  |  | Dissolved organic carbon | -10.4 |  |
| JDS2-PA | Observed ASVs | 42 | Water temperature | 14.0 | 0.22* |
|  |  |  | pH | -1.81 x 10^2^ |  |
|  |  |  | Conductivity | -7.81 x 10^-1^ |  |
|  |  |  | Dissolved organic carbon | 16.0 |  |
|  |  |  | Total suspended solids | -1.21 |  |
|  |  |  | Nitrate | 1.17 x 10^2^* |  |
| JDS3 | Observed ASVs | 43 | Water travel time | -7.82 x 10^-2^*** | 0.64*** |
|  |  |  | Conductivity | -0.93*** |  |
|  |  |  | Dissolved oxygen | -18.3** |  |
|  |  |  | Dissolved organic carbon | 24.1** |  |
|  |  |  | Total suspended solids | 1.64*** |  |

**Table S2** Summary statistics on covariation between environmental variables and the projections of bacterioplankton community samples in the Bray-Curtis based non-metric multidimensional scaling (NMDS) (**Fig. 3**). Values of covariation were calculated using the “envfit” function included in the R-package “vegan”, fitting environmental vectors onto an ordination (Oksanen et al., 2013). Significance codes: *** *P* ≤ 0.001, ** *P* ≤ 0.01, * *P* ≤ 0.05. † values reported in (Savio et al., 2015).

|  |  | **Coefficient of determination (*R*^2^)** | | |
| --- | --- | --- | --- | --- |
| **Parameter class** | **Parameter** | **JDS3** | **JDS2 FL** | **JDS2 PA** |
| Hydrology | Distance to river mouth [river km] | 0.91*** | **†** 0.84*** | **†** 0.83*** |
|  | Catchment area [km^2^] | 0.89*** | **†** 0.77*** | **†** 0.80*** |
|  | Cumulative dendritic stream lengths [km]* | 0.89*** | **†** 0.77*** | **†** 0.81*** |
|  | Median dendritic stream length [km]* | 0.85*** | **†** 0.75*** | **†** 0.77*** |
|  | Mean flow velocity [m/s] | 0.27** | 0.11 | 0.08 |
|  | Discharge (Q) [m^3^/s] | 0.49*** | 0.72*** | 0.67** |
|  | Water travel time | 0.91*** | 0.75*** | 0.64*** |
| Physico-chemistry | Conductivity | 0.45*** | 0.31** | 0.18* |
|  | Water temperature | 0.20** | 0.23** | 0.04 |
|  | pH | 0.02 | 0.15 | 0.11 |
|  | Dissolved oxyygen | 0.13* | 0.07 | 0.04 |
|  | Total suspended solids (TSS) | 0.26*** | **†** 0.14* | **†** 0.35*** |
| Nutrients | NO_3_^-^ | 0.71*** | **†** 0.68*** | **†** 0.54*** |
|  | PO_4_^3-^ | 0.12 | 0.17 | 0.11 |
|  | Total P | 0.27*** | 0.04 | 0.08 |
|  | DOC | 0.22* | 0.02 | 0.13 |
| Indicators | Chlorophyll a | 0.39*** | **†** 0.02 | **†** 0.40*** |
|  | Faecal indicator *E. coli* | 0.05 | 0.29** | 0.41*** |

* Dendritic stream length refers to the measurement of the distance along the channel of streams within a dendritic drainage basin, a common drainage pattern that resembles tree branches. The median dendritic stream length is the median length for a given stream order. The cumulative dendritic stream length is the total length of all streams in the catchment.

**Table S3.** Detailed information on ASVs showing high growth rates for the particle-associated size fraction of JDS2 (JDS2-PA), the free-living size fraction of JDS2 (JDS2-FL) and JDS3. The maximum cell production rate is the steepest slope (highest derivative) of the generalized additive model (Fig. 3, function gam of the R package mgcv). The max. growth rate is highest among the site-to-site growth rates, calculated from the absolute abundance at the upstream and downstream site, and the travel time between them.

| ASV | | JDS3 | | | | JDS2 - FL | | | | JDS2 - PA | | | |
| --- | --- | --- | --- | --- | --- | --- | --- | --- | --- | --- | --- | --- | --- |
|  |  | **Min. absolute abundance [10^6^ cells/L]** | **Max. absolute abundance [10^6^ cells/L]** | **Max. cell production rate [10^6^ cells/L/h]** | **Max. cell division rate [doublings/d]** | **Min. absolute abundance [10^6^ cells/L]** | **Max. absolute abundance [10^6^ cells/L]** | **Max. cell production rate [10^6^ cells/L/h]** | **Max. cell division rate [doublings/d]** | **Min. absolute abundance [10^6^ cells/L]** | **Max. absolute abundance [10^6^ cells/L]** | **Max. cell production rate [10^6^ cells/L/h]** | **Max. cell division rate [doublings/d]** |
| ASV_3 | **hgcI_clade [Actinobacteriota]** | 99 | 2338 | 8.42 | 4.08 | 8 | 109 | 0.10 | 11.9 | 0 | 140 | 0.20 | 14.5 |
| ASV_5 | **hgcI_clade [Actinobacteriota]** | 39 | 1567 | 6.01 | 3.53 | 3 | 116 | 0.52 | 13.3 | 0 | 148 | 0.19 | 32.7 |
| ASV_15 | **hgcI_clade [Actinobacteriota]** | 0 | 1487 | 3.57 | 17.1 | 0 | 55 | 0.10 | 4.35 | 0 | 83 | 0.11 | 13.5 |
| ASV_17 | **hgcI_clade [Actinobacteriota]** | 0 | 1167 | 3.77 | 4.03 | 0 | 60 | 0.05 | 14.7 | 0 | 60 | 0.11 | 19.0 |
| ASV_21 | **CL500-29_marine_group [Actinobacteriota]** | 0 | 1714 | 4.10 | 2.44 | 0 | 107 | 0.31 | 1.89 | 0 | 98 | 0.23 | 9.33 |

**Table S4.** Detailed information on ASVs showing high loss rates for the particle-associated size fraction of JDS2 (JDS2-PA), the free-living size fraction of JDS2 (JDS2-FL) and JDS3. The maximum loss rate is the steepest negative slope (lowest derivative) of the generalized additive model (Fig. 3, function gam of the R package mgcv).

| ASV | | JDS3 | | | JDS2 - FL | | | JDS2 - PA | | |
| --- | --- | --- | --- | --- | --- | --- | --- | --- | --- | --- |
|  |  | **Max. absolute abundance [10^6^ cells/L]** | **Min. absolute abundance [10^6^ cells/L]** | **Max. loss rate [10^6^ cells/L/h]** | **Max. absolute abundance [10^6^ cells/L]** | **Min. absolute abundance [10^6^ cells/L]** | **Max. loss rate [10^6^ cells/L/h]** | **Max. absolute abundance [10^6^ cells/L]** | **Min. absolute abundance [10^6^ cells/L]** | **Max. growth rate [10^6^ cells/L/h]** |
| ASV_25 | **Limnohabitans [Proteobacteria]** | 224 | 0 | -1.81 | 35 | 0 | -0.05 | 30 | 0 | -0.03 |
| ASV_27 | **hgcI_clade [Actinobacteriota]** | 299 | 0 | -2.40 | 30 | 0 | -0.12 | 54 | 0 | -0.04 |
| ASV_37 | **Sphingorhabdus [Proteobacteria]** | 186 | 0 | -1.22 | 58 | 0 | -0.06 | 47 | 0 | -0.06 |
| ASV_55 | **Sediminibacterium [Bacteroidota]** | 160 | 0 | -0.76 | 24 | 0 | -0.02 | 21 | 0 | -0.03 |
| ASV_71 | **Limnohabitans [Proteobacteria]** | 84 | 0 | -0.66 | 11 | 0 | -0.03 | 10 | 0 | -0.02 |
| ASV_73 | **CL500-29_marine_group [Actinobacteriota]** | 154 | 0 | -0.17 | 15 | 0 | -0.01 | 23 | 0 | -0.02 |

**SI References**

Oksanen, J., Blanchet, F.G., Kindt, R., Legendre, P., Minchin, P.R., O’hara, R., Simpson, G.L., Solymos, P., Stevens, M.H.H. and Wagner, H. 2013. Package ‘vegan’. Community ecology package, version 2(9).

Savio, D., Sinclair, L., Ijaz, U.Z., Parajka, J., Reischer, G.H., Stadler, P., Blaschke, A.P., Blöschl, G., Mach, R.L., Kirschner, A.K.T., Farnleitner, A.H. and Eiler, A. 2015. Bacterial diversity along a 2600 km river continuum. Environmental Microbiology 17(12), 4994-5007.
